# Integrative metabolomics and molecular networking reveal the progressive metabolic continuum of resin formation in *Dracaena cochinchinensis* wood

**DOI:** 10.3389/fmolb.2026.1795393

**Published:** 2026-05-28

**Authors:** Meijia Chen, Yunfei Cui, Jinhua Su, Jiahui Ren, Yu Zhu, Jinhui Wang, Guang Li

**Affiliations:** 1 College of Pharmacy, Heilongjiang University of Chinese Medicine, Harbin, China; 2 Yunnan Branch, Institute of Medicinal Plant Development, Chinese Academy of Medical Sciences & Peking Union Medical College, Xishuangbanna, China; 3 Yunnan Key Laboratory of Southern Medicine Utilization, Xishuangbanna, China; 4 Xishuangbanna Institute for Drug Control, Jinghong, China; 5 Traditional Chinese Medicine Hospital of Ya’an, Yaan, China; 6 Xishuangbanna Dai Medicine Research Institute Co., Ltd., Jinghong, Yunnan, China

**Keywords:** Dracaena cochinchinensis, Feature-Based Molecular Networking (FBMN), metabolic reprogramming, phytochemical mobilization, stress response, therapeutic compounds, wood morphotypes

## Abstract

**Introduction:**

The resinification of Dracaena cochinchinensis wood represents a sophisticated physiological reprogramming triggered by environmental stress, culminating in the production of the high-value “dragon’s blood” resin.

**Methods:**

In this study, we systematically characterized the chemical space of three representative wood morphotypes—hollow cork cambium (P), whole-body resin-containing (MZ), and resin-secreting aggregated (LZ)—to decipher the molecular mechanisms underpinning resinogenesis within the xylem matrix. Integrating UPLC-Q-TOF-MS/MS with Feature-Based Molecular Networking (FBMN), we overcame annotation bottlenecks inherent in complex woody tissues and successfully annotated 299 specialized metabolites, including flavonoids, phenylpropanoids, steroids, and lipids.

**Results:**

Multivariate statistical analysis revealed significant metabolic shifts across these wood morphotypes, reflecting distinct biological strategies: the P form prioritized phenylpropanoid biosynthesis (e.g., sinapyl alcohol and coniferaldehyde) for structural barrier reinforcement of the wood cell walls; the MZ morphotype accumulated steroids and fatty acids to maintain membrane integrity during chronic adaptation; and the LZ morphotype exhibited a defensive burst of flavonoids and terpenoids, typical of acute stress responses.

**Discussion:**

Collectively, these findings support a putative “progressive metabolic continuum” model (P→MZ→LZ), illustrating a hypothesized metabolic gradient and a dynamic shift in resource allocation from physical repair to chronic adaptation and finally to acute chemical defense. This study provides a comprehensive phytochemical framework for understanding specialized metabolite mobilization in resinous wood and establishes an efficient metabolomic workflow for the quality evaluation and sustainable utilization of woody medicinal resources.

## 1 Introduction

Resin from *Dracaena cochinchinensis* is the definitive botanical source of “Dragon’s Blood” (Draconis Sanguis), a prestigious traditional medicine utilized for over 1,500 years to enhance hemorheology, arrest hemorrhage, and accelerate tissue regeneration (Zhu et al., 2007; Lang et al., 2020). Modern pharmacological research has elucidated that its therapeutic efficacy is governed by a diverse repertoire of bioactive specialized metabolites—predominantly flavonoids and phenolic acids—which possess potent anti-inflammatory, anti-atherosclerotic, and wound-healing properties (Xu et al., 2022; Zhang et al., 2022; Wen et al., 2016; Xin et al., 2011). Despite its profound clinical significance, the natural populations of *Dracaena cochinchinensis* are increasingly threatened by overexploitation and habitat fragmentation. Therefore, deciphering the molecular mechanisms governing the biosynthesis and mobilization of these therapeutic compounds within the complex woody matrix is imperative for both the standardized utilization and the conservation of this endangered resource.

The biogenesis of resin in *Dracaena cochinchinensis* is a quintessential manifestation of the plant’s plastic defensive response. Upon mechanical injury or opportunistic fungal infection, including Fusarium graminearum and Fusarium moniliforme (Wang et al., 2011), the plant initiates a massive secondary metabolic reprogramming. Defensive phytochemicals, including flavonoids and terpenoids, accumulate and polymerize within xylem cells to form a physical and chemical barrier. However, this defensive process leads to significant chemical heterogeneity across different wood morphotypes, resulting in inconsistent resin quality. Understanding the metabolic shifts during the transition from healthy wood to highly resinous tissue is crucial for identifying the molecular markers of high-quality “Dragon’s Blood.”

To capture the complexity of the *Dracaena* metabolome, high-resolution analytical strategies are required. While traditional platforms like GC-MS and NMR provide valuable insights (Patel et al., 2021; Delfin et al., 2019). UPLC-Q-TOF-MS/MS offers superior sensitivity and throughput for non-volatile small molecules (Ma and Qi, 2021). To overcome the bottleneck of structural annotation in complex plant matrices, Feature-Based Molecular Networking (FBMN) via the GNPS platform has emerged as a transformative tool (Nothias et al., 2020). By utilizing cosine similarity algorithms to cluster compounds with analogous fragmentation patterns, FBMN enables the systematic annotation of unknown derivatives and molecular families that were previously overlooked (Li et al., 2024; Li et al., 2023).

**FIGURE 1:**
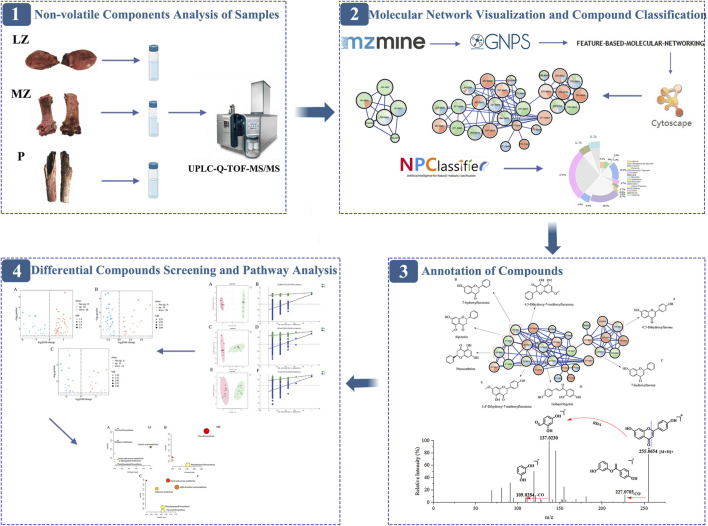
Schematic representation of the workflow of the study.

In this study, we investigated the non-volatile secondary metabolome of three representative *Dracaena cochinchinensis* wood morphotypes: hollow cork cambium (P), whole-body resin-containing wood (MZ), and resin-secreting aggregated wood (LZ). By integrating untargeted metabolomics with multivariate statistics and FBMN, we aimed to map the spatial phytochemical landscape of the wood matrix and propose a hypothesized “progressive metabolic continuum” model to elucidate the potential resource allocation strategies during resinogenesis. This model elucidates the resource allocation strategies of *Dracaena cochinchinensis* during resinogenesis, providing a theoretical framework for the sustainable development and molecular quality evaluation of this high-value medicinal resource. The overall research workflow is illustrated in Figure 1.

## 2 Materials and methods

### 2.1 Instruments, samples, and reagents

Chromatographic separation was performed using an X500B ultra-performance liquid chromatography-quadrupole time-of-flight mass spectrometry (UPLC-Q-TOF-MS/MS) system (AB SCIEX, United States). Other equipment included an SB-1200 ultrasonic cleaner (Ningbo Scientz Biotechnology Co., Ltd., China), a DV215CD 1/100,000 electronic analytical balance (OHAUS, United States), an FW80 high-speed universal crusher (Tianjin Taiste Instrument Co., Ltd., China), and a Sigmal-14 benchtop low-speed centrifuge (Branch Xingyu Technology Development Co., Ltd., China).

LC-MS grade methanol and acetonitrile were purchased from Thermo Fisher Scientific (Waltham, MA, United States). Formic acid (LC-MS grade) was obtained from Sigma-Aldrich (St. Louis, MO, United States). Ultrapure water was prepared using an ELGA LabWater system (UK). Other reagents were of analytical grade.

The resin-containing wood samples of *Dracaena cochinchinensis* were categorized into three representative morphotypes. To ensure statistical robustness, eleven biological replicates (n = 11) were collected for each group (P, MZ, and LZ). Samples were harvested from wild trees using sterilized stainless-steel saws. To minimize post-harvest metabolic alterations, the collected woody tissues were immediately snap-frozen in liquid nitrogen and subsequently stored at −80 °C until extraction. The abbreviations P, MZ, and LZ are derived from the Pinyin of their traditional Chinese commercial classifications, which are defined strictly by macroscopic resin morphology: “Pi” (P, referring to the hollow cork cambium morphotype with only cortical resin deposition), “ManZhi” (MZ, referring to the whole-body resin-containing wood morphotype with uniform resin distribution in the xylem), and “LiuZhi” (LZ, referring to the nodule-like aggregated resin-secreting wood morphotype with massive concentrated resin accumulation). These three morphotypes were specifically selected because they represent the most typical, phenotypically distinct, and commercially significant stages of natural resin accumulation in the traditional medicine market, covering a continuous gradient from low to high resin content.

All samples were wild-harvested, naturally resinous materials provided by Xishuangbanna Jianlong Pharmaceutical Co., Ltd. and authenticated by experts. Because these materials were sourced from natural habitats, exact historical metadata regarding the age of the mother trees, highly specific micro-environmental conditions, and the precise initial natural infection triggers were untraceable. Consequently, samples were strictly grouped based on their physiological morphotypes. Detailed sample information, including geographic origin and batch numbers, is provided in Table 1.

**TABLE 1:** Sample information of resin-containing wood in *Dracaena cochinchinensis* from different appearance.

NO.	Batch	Source	Morphotype	NO.	Batch	Source	Morphotype
1	200,513	Northern Myanmar	P	18	230,128	Central Myanmar	MZ
2	200,513	Northern Myanmar	P	19	230,128	Central Myanmar	MZ
3	200,513	Northern Myanmar	P	20	230,128	Central Myanmar	MZ
4	200,513	Northern Myanmar	P	21	230,128	Central Myanmar	MZ
5	200,513	Northern Myanmar	P	22	230,128	Central Myanmar	MZ
6	200,513	Northern Myanmar	P	23	200,513	Myanmar	LZ
7	200,513	Northern Myanmar	P	24	200,513	Myanmar	LZ
8	200,513	Northern Myanmar	P	25	200,513	Myanmar	LZ
9	200,513	Northern Myanmar	P	26	200,513	Myanmar	LZ
10	200,513	Northern Myanmar	P	27	230,128	Myanmar	LZ
11	200,513	Northern Myanmar	P	28	230,128	Myanmar	LZ
12	200,513	Central Myanmar	MZ	29	230,128	Myanmar	LZ
13	200,513	Central Myanmar	MZ	30	230,128	Myanmar	LZ
14	200,513	Central Myanmar	MZ	31	230,128	Myanmar	LZ
15	230,128	Central Myanmar	MZ	32	230,128	Myanmar	LZ
16	230,128	Central Myanmar	MZ	33	230,128	Myanmar	LZ
17	230,128	Central Myanmar	MZ				

### 2.2 Methods

#### 2.2.1 Sample extraction

The sample preparation followed standard protocols. Briefly, the medicinal material was crushed and sieved through a No. 3 sieve. A precise amount of 2.0 g of powder was weighed and placed into a 100 mL conical flask, to which 50 mL of 95% ethanol was added. The mixture was subjected to ultrasonic extraction for 30 min. Subsequently, the flask was centrifuged at 10,000 rpm for 10 min. The supernatant was filtered through a 0.22 μm membrane, and the filtrate was stored at 4 °C for UPLC-Q-TOF-MS/MS analysis.

#### 2.2.2 HPLC conditions

Separation was achieved on an Acquity UPLC BEH C18 column (150 mm × 2.1 mm, 1.7 μm). The mobile phase consisted of 0.1% formic acid in water (A) and acetonitrile containing 0.1% formic acid (v/v) (B). The gradient elution program was as follows: 0–2 min, 95% A; 2–3 min, 95%–60% A; 3–7 min, 60%–40% A; 7–10 min, 40%–20% A; 10–13 min, 20%–0% A; 13–15 min, 0%–95% A; 15–20 min, 95% A. The column temperature was maintained at 40 °C, the flow rate was 0.3 mL/min, and the injection volume was 2 μL.

#### 2.2.3 MS conditions

Electrospray ionization (ESI) was operated in both positive and negative ion modes. The scanning range was set to *m/z* 100–1200 (primary) and *m/z* 50–1200 (secondary) in both positive and negative ESI modes. Key parameters were: ionization voltage (ISVF) +5500/-5500 V; source temperature (TEM) 550 °C; ion source gas 1 (GS1) and gas 2 (GS2) 60 psi; curtain gas (CUR) 35 psi. The collision energy (CE) was set to 35 ± 15 eV to obtain informative MS/MS spectra for feature annotation.

#### 2.2.4 Data processing and FBMN construction

To ensure system stability and data reproducibility, quality control (QC) samples were prepared by pooling equal aliquots (50 μL) from all biological samples. The QC samples were injected every six analytical samples throughout the run. Additionally, blank samples (95% ethanol) were analyzed at the beginning and end of the batch to identify and eliminate background noise and carryover contamination. Peak areas were normalized to the total ion intensity of each chromatogram before multivariate analysis.

Raw mass spectrometry data (.wiff) were converted to.mzML format using MSConvert (ProteoWizard). Data processing, including peak detection, deconvolution, and alignment, was performed using MZmine 2.53. The processed feature table and MS/MS spectral data (.mgf) were exported and uploaded to the Global Natural Product Social Molecular Networking (GNPS) platform for Feature-Based Molecular Networking (FBMN) analysis. The precursor ion mass tolerance and MS/MS fragment ion tolerance were both set to 0.02 Da. A cosine score threshold of 0.7 and a minimum of six matched peaks were required for edge creation. The resulting molecular network was visualized using Cytoscape 3.10.1. Metabolite annotation was performed following the Metabolomics Standards Initiative (MSI) guidelines. Raw MS/MS data were matched against public databases, including GNPS and MassBank. In the absence of reference standards, all compounds were assigned to MSI Level 2 (putatively annotated compounds). Annotation confidence was further enhanced by manually verifying characteristic fragmentation patterns and neutral losses against literature data specifically for *Dracaena* species.The positive ion mode dataset generated by FBMN can be accessed via the following link: https://gnps.ucsd.edu/ProteoSAFe/status.jsp?task=7e7c68584bdc4f9ba64786aa65d9a2b4; The negative ion mode dataset can be accessed via the following link: https://gnps.ucsd.edu/ProteoSAFe/status.jsp?task=71a7007093c74a58972b82d45ed1761e. Visualized the created network using Cytoscape 3.10.1.

#### 2.2.5 Multivariate statistical analysis

The resulting data matrix (peak areas) was imported into MetaboAnalyst 6.0 (https://www.metaboanalyst.ca/) for normalization and multivariate analysis, including Principal Component Analysis (PCA) and Partial Least Squares Discriminant Analysis (PLS-DA). Orthogonal Partial Least Squares Discriminant Analysis (OPLS-DA) was verified using SIMCA 14.1 software (Umetrics, Sweden) to maximize group separation. Differential metabolites were screened based on Variable Importance in Projection (VIP) > 1 and p < 0.05 (Student’s t-test).

## 3 Results and discussion

### 3.1 UPLC-Q-TOF-MS/MS analysis of three morphotypes of *Dracaena cochinchinensis*

To investigate the compositional variations among different morphotypes of resin-containing wood in *Dracaena cochinchinensis*, a non-targeted UPLC-Q-TOF-MS/MS analysis was conducted on three distinct morphological samples: the hollow cork cambium sample (P), the whole-body resin-containing sample (MZ), and the resin-secreting aggregated sample (LZ). A comprehensive analysis was performed on all samples in both positive and negative ion modes, resulting in the total ion current spectra for LZ, MZ, and P, as depicted in Supplementary Figure S1. There were observable differences in the number and intensity of the peak groups across the samples, indicating significant variations in the types and concentrations of metabolites present in the resin-containing wood of *Dracaena cochinchinensis* across these three morphotypes.

### 3.2 Exploring the chemical space characteristics of three morphological groups of *Dracaena cochinchinensis* through FBMN

This study utilized Feature-Based Molecular Networking (FBMN) technology to analyze the chemical compositions of various *Dracaena cochinchinensis* samples. In the positive ion mode, 44,008 metabolite features were detected, resulting in the formation of 12,757 molecular families (defined as those with ≥2 nodes) through FBMN analysis, as depicted in Supplementary Figure S2. In the negative ion mode, 23,040 metabolite features were detected, leading to the annotation of 5,774 molecular families (Supplementary Figure S3). The nodes were visually represented by color-coded pie charts, indicating the relative abundance of metabolites across three groups: red for MZ, green for LZ, and blue for P.

Notably, larger nodes correspond to compounds annotated within the GNPS database. After rigorous filtering to exclude potential contaminants (e.g., plasticizers and siloxanes), a cumulative total of 299 unique compounds were annotated. Detailed information for all annotated metabolites, including theoretical *m/z*, observed *m/z*, error (ppm), and MS/MS fragments, is provided in Supplementary Table S1.

The NPClassifier tool was employed to categorize the metabolites of *Dracaena cochinchinensis* into distinct chemical groups, including flavonoids and their derivatives, lipids, phenolic derivatives, steroids, carbohydrates and glycosides, and so on. As shown in Supplementary Figure S4, flavonoids and lipids compounds hold primary significance in the chemical profile.

### 3.3 Structure analysis and pharmacological action of metabolites

The FBMN algorithm aided in the clustering of compounds with similar MS2 fragmentation patterns into molecular families. An in-depth manual analysis of compound structures was performed to investigate the structural interconnections among components within the flavonoid, lipid, phenolic acid, and terpenoid molecular families.

#### 3.3.1 Annotations of flavonoid molecular families

**FIGURE 2:**
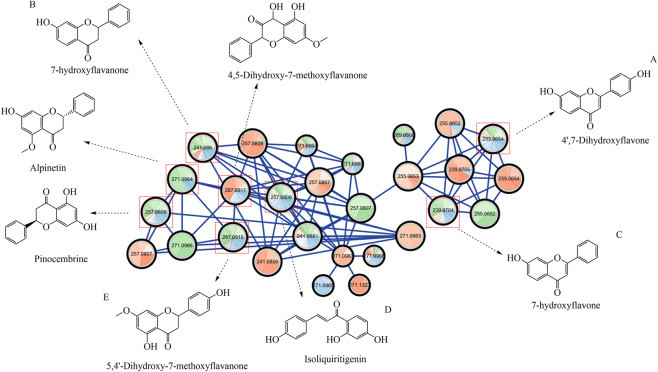
Visualization of flavonoid molecular families across wood morphotypes. Pie chart colors represent the relative abundance of metabolites in MZ (red), LZ (green), and P (blue) morphotypes.

Flavonoids represent the primary chemical constituents of *Dracaena cochinchinensis* and exhibit significant pharmacological activity. The structural diversity of these compounds has been shown to directly influence the quality of medicinal materials. This study focused on the flavonoid molecule family (Figure 2) in alignment with the FBMN molecular network clustering results. High-resolution mass spectrometry fragmentation analysis was utilized, alongside characteristic fragment ions and neutral loss patterns, to systematically elucidate the fragmentation pathways and structural characteristics of five key flavonoid compounds. This methodology provided a foundational basis for interpreting the functional implications of metabolic differences. Flavonoid compounds possess a core C15 benzopyranone structure, which is further categorized into various subclasses based on the substituents present on the A and B rings, the saturation level of the C ring, and the sites of glycosylation. The predominant cleavage mechanism is Retro-Diels-Alder (RDA) cleavage, which generates characteristic fragments from the A/B rings (e.g., *m/z* 137, 129). In the positive ion mode, protonation sites are preferentially situated at the carbonyl or hydroxyl groups of the C ring, resulting in major fragments such as decarboxylation (−44 Da), decarbonylation (−28 Da), and dehydration (−18 Da) products (Wojtanowski and Mroczek, 2020; Jiang and Gates, 2024; March and Brodbelt, 2008).

**FIGURE 3:**
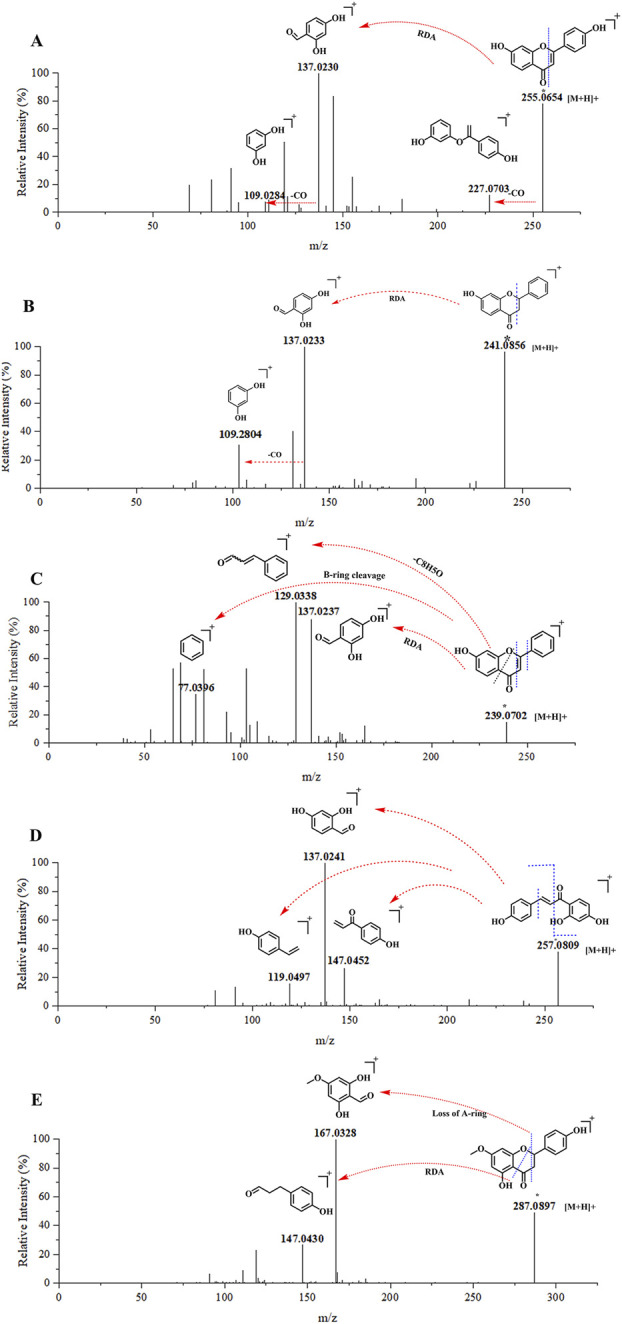
Structural annotation of key flavonoid compounds with therapeutic interest. MS/MS spectra were acquired in positive ion mode with a collision energy of 35 ± 15 eV. Parent ions ([M + H]^+^) are indicated, and characteristic structural fragmentation patterns are mapped with corresponding high-precision *m/z* values (four-decimal precision) for key diagnostic fragment peaks. **(A)** 4,7-Dihydroxyflavone; **(B)** 7-Hydroxyflavanone; **(C)** 7-Hydroxyflavone; **(D)** Isoliquiritigenin; **(E)** 5,4′-dihydroxy-7-methoxyflavanone.

The MS1 analysis of compound A reveals a precursor ion at *m/z* 255.0654 [M + H]^+^, suggesting a molecular formula of C_15_H_10_O_4_^+^. The fragmentation pathway, depicted in Figure 3A, demonstrates that in positive ion mode, C_15_H_10_O_4_ undergoes RDA fragmentation, yielding C_7_H_5_O_3_^+^ (*m/z* 137.0230), which subsequently undergoes secondary carbon monoxide (CO) loss to form C_6_H_5_O_2_^+^ (*m/z* 109.0284). Additionally, the CO loss from C_15_H_10_O_4_^+^ results in the formation of C_14_H_10_O_3_^+^ (*m/z* 227.0703). Based on these fragmentation patterns and corroborated by databases such as HMDB, ChEBI, DrugBank, and PubChem, compound A was annotated as 4,7-dihydroxyflavone.

The molecular ion of compound B was observed at *m/z* 241.0856 [M + H]^+,^ suggesting a molecular formula of C_15_H_12_O_3_. The fragmentation pathway of this ion is illustrated in Figure 3B. The molecular ion undergoes RDA fragmentation, leading to the cleavage of the 7-hydroxymethyl group (C_7_H_5_O_3_^+^) at *m/z* 137.0233. Subsequently, the ion at *m/z* 137.0233 undergoes further fragmentation with the loss of carbon dioxide (28 Da), resulting in the formation of a phenyl ketone ion (C_6_H_5_O_2_^+^, *m/z* 109.0241). Based on these fragmentation pathways, and corroborated by data from HMDB, KNApSAcK, ChEBI, DrugBank, and other pertinent literature sources (Sun et al., 2009; Khalifa et al., 2023), it is hypothesized that the compound is 7-hydroxyflavanone.

The MS1 of compound C reveals its precursor ion at *m/z* 239.0704 [M + H]^+^, with the proposed molecular formula C_15_H_10_O_3_. The fragmentation pathway is depicted in Figure 3C initially, the 7-hydroxy group attacks the carbonyl group, leading to cleavage of the C ring and the formation of an indanone structure, resulting in C_9_H_5_O^+^ (*m/z* 129.0338). Subsequently, ring cleavage occurs, accompanied by decarboxylation or hydrogen transfer, yielding C_7_H_5_O_3_^+^ (*m/z* 137.0237). The B ring is further cleaved to produce the benzyl ion C_6_H_5_^+^ (*m/z* 77.0396). Based on these fragmentation pathways, along with corroborative data from databases such as MassBank, HMDB, COCONUT, and existing literature (Soliman et al., 2021), the compound is tentatively annotated as 7-hydroxyflavone.

The MS1 analysis of compound D reveals its precursor ion at *m/z* 257.0809 [M + H]^+^, corresponding to the proposed molecular formula C_15_H_12_O_4_. The fragmentation pathway of this compound is depicted in Figure 3D. Key stages in this process include the retro-Diels-Alder (RDA) cleavage of the A ring, the cleavage of the dihydrofuranone ring (C ring), and the formation of a stable benzofuranone ion (C_7_H_5_O_3_^+^) at *m/z* 137.0241, as well as C8H7O^+^ at *m/z* 119.0497, originating from the A ring with a 4′-hydroxy substitution. Additionally, a benzyl ketone ion (C_8_H_7_O_2_^+^) at *m/z* 147.0452 can be formed through the rearrangement of the A ring hydroxyl group. Based on these fragmentation pathways, alongside database references such as HMDB, ChEBI, and DrugBank, as well as literature reports (Xie et al., 2014; Xue et al., 2025), it is hypothesized that compound D is isoliquiritigenin. Importantly, we carefully differentiated isoliquiritigenin from its flavanone isomer, liquiritigenin. While GNPS matching may assign similar scores to both isomers due to identical precursor ions (*m/z* 257.0809), their structural divergence leads to specific MS/MS behaviors. The robust presence of the diagnostic fragment at *m/z* 119.0497 (C_8_H_7_O^+^), generated by the characteristic cleavage of the open-chain chalcone skeleton, strongly justifies the annotation of compound D as isoliquiritigenin. In contrast, flavanones like liquiritigenin typically undergo different fragmentation pathways due to their closed C-ring structure, which precludes the formation of this specific diagnostic fragment. Furthermore, it should be noted that redundant nodes are occasionally generated by the FBMN algorithm—such as the unannotated red node sharing the identical precursor ion at *m/z* 257.0809 (RT: 6.42 min) shown in Figure 2. Due to its extremely low intensity (low abundance) and the lack of high-quality MS/MS fragmentation data, this feature was identified as a redundant artifact or computational noise rather than a distinct specialized metabolite. Consequently, this node was systematically de-replicated and filtered out during our subsequent manual curation. This stringent data-cleaning step ensures that only unique, high-confidence metabolites are retained in the final curated dataset (Supplementary Table S1).

The MS1 of compound E shows its precursor ion as *m/z* 287.0897[M + H]^+^, with the proposed molecular formula C_16_H_14_O_5_. The mass spectrometry fragmentation pathway is illustrated in Figure 3E: the B-ring carbonyl rearranges to form C_8_H_7_O_4_^+^ (*m/z* 167.0328); the rearrangement of the A ring with the C2-C3 portion yields a benzoate cation derivative (C_9_H_7_O_2_^+^) (*m/z* 147.0430). In view of the above fragmentation pathways, in conjunction with the HMDB and PubChem databases, the compound in question is tentatively annotated as 5,4′-Dihydroxy-7-methoxyflavanone.

The integration of multi-level mass spectrometry fragment data with molecular network clustering has facilitated the annotation of key flavonoid metabolites, including 4,7-dihydroxyflavone (A), 7-hydroxyflavanone (B), 7-hydroxyflavone (C), isoliquiritigenin (D), and 5,4′-Dihydroxy-7-methoxyflavanone (E). These compounds are crucial for *Dracaena cochinchinensis*’s stress defense and their distribution supports further metabolic function analysis. Additionally, they exhibit diverse pharmacological effects. For instance, 4,7-dihydroxyflavone can form N-O hydrogen bonds with HIF-1α via THR-327 residues, potentially alleviating anemia (Qu et al., 2025). Moreover, 7-hydroxyflavone (Ga and A, 2025) has been reported to show potential in treating protein diseases by binding to Hb near high-affinity amino acid sequences, protecting it from crowding-induced denaturation. Moreover, it has been demonstrated to exhibit cardioprotective and antioxidant properties, suggesting a potential therapeutic advantage in addressing cardiotoxicity induced by the first-line chemotherapy agent doxorubicin (Sangweni et al., 2023). Additionally, 7-Hydroxyflavone has been reported as a promising pharmaceutical candidate for the treatment of osteoporosis. This study hypothesizes that the prevention of dexamethasone-induced osteoporosis is mediated through the modulation of the GATA-3/Caspase-3/NF-KB pathway. Beyond these attributes, the compound displays a range of pharmacological activities, including antioxidant, anti-inflammatory, and vascular protective effects (Sengupta et al., 2017; Qi et al., 2024; Wan et al., 2019; Calderone et al., 2004).As a bioactive compound, isoliquiritigenin has demonstrated neuroprotective properties and potential therapeutic benefits for neurodegenerative diseases such as Alzheimer’s Disease (AD) and Parkinson’s Disease (PD). The principal mechanism underlying the effects of isoliquiritigenin is its ability to act as a neuroprotective agent and antioxidant, thereby reducing excessive reactive oxygen species (ROS)-mediated neuronal damage in the brain (Shi et al., 2020). To date, 5,4′-Dihydroxy-7-methoxyflavanone has been documented to exhibit a limited range of pharmacological effects. Nonetheless, research has demonstrated its anti-inflammatory (Soromou et al., 2012) and anti-malarial properties (Ahmed et al., 2001), among other effects.

The structural diversity and significant enrichment of these flavonoids in the LZ wood morphotype provide a definitive molecular basis for its superior pharmacological quality. As the primary bioactive constituents, these compounds contribute to the systemic anti-inflammatory, anti-thrombotic, and wound-healing efficacy traditionally associated with high-grade Dragon’s Blood, highlighting the therapeutic interest of the LZ form in pharmaceutical development.

#### 3.3.2 Annotations of the phenolic compound family

**FIGURE 4:**
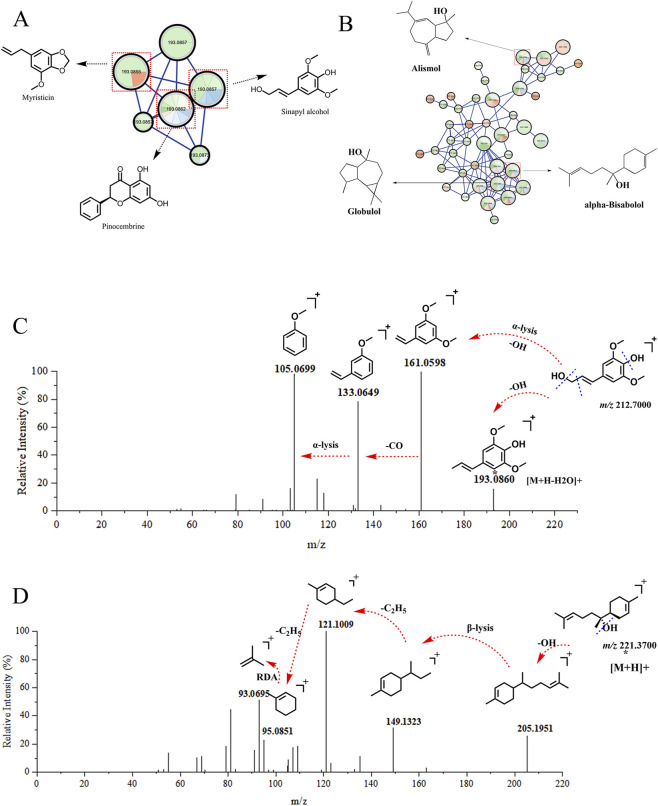
Molecular networking and structural characterization of phenolic and terpenoid families. MS/MS spectra were acquired under identical conditions (collision energy: 35 ± 15 eV). The y-axes are denoted as “Relative Intensity (%)” to demonstrate fragmentation abundance, with key precursor and product ions precisely labeled. **(A)** Phenolic molecular families; **(B)** Terpenoid molecular families; **(C)** Structural annotation of sinapyl alcohol; **(D)** Structural annotation of α-Bisabolol.

The molecular family of phenolic compounds is shown in Figure 4A. The MS1 of compound A indicates that its quasi-molecular ion is *m/z* 193.0860 [M + H-H_2_O]^+^, with a fitted molecular formula of C_11_H_14_O_4_. The fragmentation pathway of this compound is illustrated in Figure 4C. The β-cleavage of the propylene alcohol side chain results in the loss of CH2OH, forming a stable phenylpropyl ion (C_10_H_11_O_3_^+^) (*m/z* 161.0598). Subsequent to this, the loss of CO results in the formation of a decarboxylation fragment (C_9_H_11_O_2_^+^) (*m/z* 133.0649). The initial step in this process is the loss of a carbon monoxide atom, which results in the formation of a phenylpropyl fragment (C_8_H_11_O^+^) or a benzyl ion (C_8_H_9_^+^) (*m/z* 105.0699). This process occurs through the direct cleavage of the benzene ring.In consideration of the aforementioned cleavage pathways, in conjunction with the HMDB, DrugBank, PubChem databases, and reference (Takahashi et al., 2011), it is hypothesized that compound A is the lignin monomer sinapyl alcohol.

Sinapic alcohol is acknowledged as a precursor in lignin biosynthesis (Yamaguchi et al., 2023). The enzymatic conversion of cellulose into lignin compounds within plant cell walls confers mechanical strength and durability to woody tissues. This lignification of cell walls serves as a crucial barrier against pathogen invasion (Zhang et al., 2025). Beyond its pivotal role in plant physiological processes, sinapic alcohol exhibits significant potential in therapeutic applications. Extensive research has demonstrated that mustard oil and its derivatives not only possess anti-inflammatory properties (Choi et al., 2004) but also mitigate the increase in vascular permeability induced by acetic acid in murine models. Additionally, they inhibit the production of NO, PGE2, and TNF-α triggered by LPS. Moreover, these compounds exhibit notable biological activities, including antioxidant effects, myocardial protection, and the enhancement of skin wound healing (Yamaguchi et al., 2023).

The annotation of lignin monomers such as sinapyl alcohol and other phenolic derivatives underscores the nutraceutical potential of the early-stage resinous wood. Beyond their role in structural barrier reinforcement, these phenolics serve as potent natural antioxidants. Their systematic mobilization in the P and MZ morphotypes suggests an strategic accumulation of compounds that may offer oxidative stress protection, further expanding the therapeutic utility of *Dracaena cochinchinensis* matrices.

#### 3.3.3 Annotation of the molecular family of terpenoids

Terpenoids have been reported as active constituents in *Dracaena cochinchinensis*. In the MS2 analysis of terpenoids, several processes are observed, including RDA cleavage, the loss of neutral molecules such as water (H_2_O, 18 Da) or carbon monoxide (CO, 28 Da), and the cleavage of saturated rings. The terpenoid molecular family is depicted in Figure 4B.

The MS1 of compound A reveals a quasi-molecular ion at *m/z* 205.1951 [M + H]^+^, with a proposed molecular formula of C_15_H_26_O. The fragmentation pathway of this compound, as elucidated through mass spectrometry, is illustrated in Figure 4D. The cyclohexane ring undergoes RDA cleavage, resulting in the formation of a stable allylic ion (C_9_H_13_^+^) at *m/z* 121.1009 and a neutral fragment C_6_H_13_O following ring cleavage. The allylic ion subsequently undergoes the loss of a vinyl group (-C_2_H_4_), leading to the formation of C_7_H_9_^+^ (*m/z* 93.0695) and a neutral fragment C2H4. Based on these cleavage pathways and corroborated by literature reports (Perbellini et al., 2004), compound A is hypothesized to be α-bisabolol.

In recent years, α-bisabolol has garnered significant interest within the scientific community. Numerous studies have demonstrated that α-bisabolol possesses the ability to modulate various signaling molecules, including IL-6, IL-1β, IL-17A, IL-23, and TNF-α, thereby exerting anti-inflammatory effects (Wu et al., 2025; Vm et al., 2025; Barreto et al., 2016). The potential therapeutic applications of α-bisabolol encompass the treatment of conditions such as psoriasis, osteoarthritis, and other related diseases. Furthermore, α-bisabolol has been shown to exhibit anticancer properties (Eddin et al., 2022) through multiple mechanisms, including the downregulation of the AKT signaling pathway, inhibition of vascular endothelial cell proliferation, and induction of apoptosis in tumor cells.

The presence of high-potency sesquiterpenoids like α-bisabolol, particularly in the resin-rich tissues, highlights the multi-component therapeutic synergy of the wood matrix. The annotation of these terpenoids as key markers reinforces the value of *Dracaena cochinchinensis* as a critical resource for extracting bioactive molecules with proven anti-inflammatory and anticancer properties, aligning with the growing demand for standardized nutraceutical discovery from complex plant matrices.

### 3.4 Multivariate statistical analysis and metabolic pathway resolution

#### 3.4.1 Pattern recognition analysis (PCA and OPLS-DA)

**FIGURE 5:**
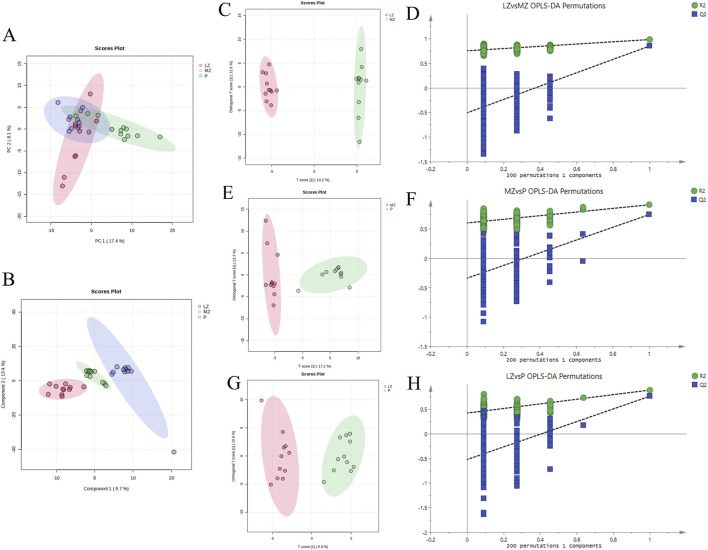
PCA, PLS-DA and OPLS-DA analyses of resin-containing wood from different morphotypes of *Dracaena cochinchinensis*. **(A)** PCA of resin-containing wood from different morphotypes of *Dracaena cochinchinensis*; **(B)** PLS-DA of resin-containing wood from different morphotypes of *Dracaena cochinchinensis*; **(C,E,G)** Two-by-two group comparisons of OPLS-DA; **(D,F,H)** Two-by-two group comparisons of OPLS-DA model permutation test plots.

To statistically determine the metabolic differences among the three morphotypes, we employed multivariate pattern recognition. First, an unsupervised Principal Component Analysis (PCA) was conducted to visualize the intrinsic structure of the data. As illustrated in Figure 5A, the PCA score plot showed a clear trend of separation. The first two principal components (PC1 and PC2) explained 25.5% of the total variance. Although there was some clustering, the partial overlap between groups suggested that unsupervised analysis alone was insufficient to fully resolve the subtle metabolic differences, likely due to the confounding effect of geographic origins. Subsequently, Partial Least Squares Discriminant Analysis (PLS-DA) was employed, which showed an improved separation trend (Figure 5B).

To maximize group separation and isolate morphotype-specific markers, we applied supervised Orthogonal Partial Least Squares Discriminant Analysis (OPLS-DA). The OPLS-DA score plots for pairwise comparisons (LZ vs. MZ, MZ vs. P, and LZ vs. P) demonstrated complete separation of the groups (Figures 5C,E,G), indicating that the metabolic signal related to wood morphology is distinct and robust. The model quality was rigorously evaluated using the goodness-of-fit parameter R^2^Y and the predictive ability parameter Q^2^. As shown in Supplementary Table S2, the R^2^Y values of the three pairwise OPLS-DA models ranged from 0.892 to 0.986, with Q^2^ values between 0.747 and 0.855, indicating that the models had high classification stability and strong predictive power. Furthermore, permutation tests (n = 200) yielded p-values <0.05 (Figures 5D,F,H), confirming that the observed separation was statistically significant and not an artifact of overfitting. These results provide strong statistical evidence that, despite the influence of geographic origin, distinct and stable metabolic fingerprints define each morphotype.

#### 3.4.2 Selection of differential metabolites and marker compounds

**FIGURE 6:**
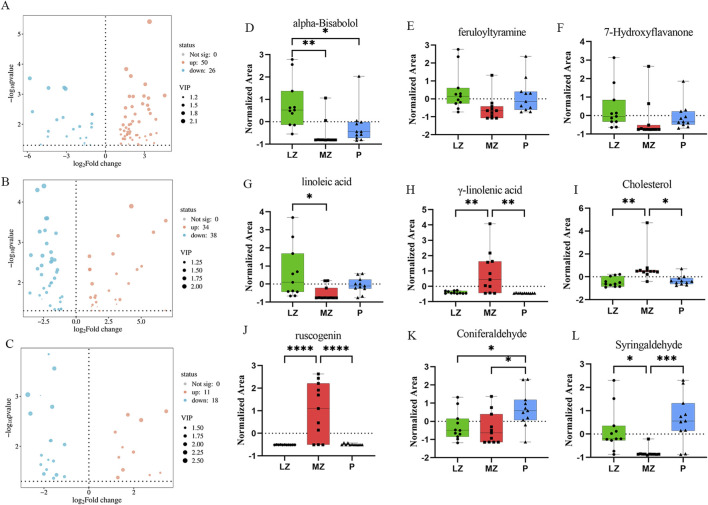
Volcano plots for two-by-two comparisons and box plots for nine differential compounds. **(A–C)** are volcano plots comparing differential compounds in pairs among three morphotypes of *Dracaena cochinchinensis* resin-containing wood; **(D–L)** are box plots; Among them are red for MZ, green for LZ and blue for P. **(D)** alpha-Bisabolol; **(E)** feruloyltyramine; **(F)** 7-Hydroxyflavanone; **(G)** linoleic acid; **(H)** γ-linolenic acid; **(I)** Cholesterol; **(J)** ruscogenin; **(K)** Coniferaldehyde; **(L)** Syringaldehyde.

Differential metabolites were screened using a strict combination of criteria: Variable Importance in Projection (VIP) > 1, |Fold Change (FC)| > 2, and a Student’s t-test p-value <0.05. The 76 metabolites were differentially accumulated between LZ and MZ (50 increased, 26 decreased), 72 between MZ and P (34 increased, 38 decreased), and 29 between LZ and P (11 increased, 18 decreased). Their distributions are visualized in the Volcano Plots (Figure 6). Hierarchical clustering analysis (Supplementary Figure S8) visually confirmed that the abundance of these metabolites varied dramatically across the three morphotypes, clustering samples into groups consistent with their morphological classifications.

Specific chemical signatures were annotated for each morphotype, reflecting distinct biological strategies. The LZ morphotype was characterized by the significant increased accumulation of flavonoids (e.g., 7-hydroxyflavone, pinocembrin, apigenin) and lipid signaling molecules such as linoleic acid. Linoleic acid is the biosynthetic precursor of jasmonic acid (JA), the central hormone in plant wound signaling. The co-accumulation of JA precursors and downstream defensive flavonoids suggests that the LZ morphotype may represent a putative state of “Acute Chemical Defense,” where resources appear to be mobilized to seal wounds and kill pathogens (Ali et al., 2017). In contrast, the MZ morphotype was distinguished by elevated levels of steroids (e.g., cholesterol, ruscogenin) and fatty acids. Unlike the rapid defense response in LZ, this profile potentially indicates an association with “Chronic Adaptation.” The accumulation of sterols and lipids is crucial for maintaining cell membrane fluidity and stability under long-term environmental stress (e.g., drought) and provides energy reserves for survival (Sonawane et al., 2016). Finally, the P morphotype showed high levels of phenylpropanoids related to lignin biosynthesis, specifically coniferaldehyde and sinapyl alcohol. These compounds are direct precursors for lignin polymerization. The abundance of these structural monomers indicates that the P morphotype prioritizes “Physical Repair”—reinforcing the cork layer and cell walls to create a physical barrier against external threats (Yoshioka et al., 2024; Yamamoto et al., 2020).

#### 3.4.3 Metabolic pathway enrichment and regulatory network reconstruction

**FIGURE 7:**
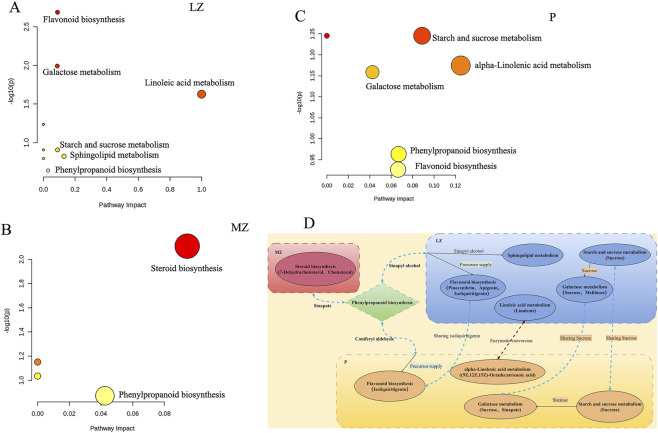
Pathway enrichment diagram of the first 25 differential compounds from the three morphotypes of *Dracaena cochinchinensis* and Metabolic pathway network diagrams of the three morphotypes of *Dracaena cochinchinensis*. **(A)** Pathway enrichment diagram of the first 25 differential compounds for the LZ morphotype. **(B)** Pathway enrichment diagram of the first 25 differential compounds for the MZ morphotype. **(C)** Pathway enrichment diagram of the first 25 differential compounds for the P morphotype. **(D)** Metabolic pathway network diagram of the three morphotypes.

To elucidate the biological mechanisms underlying these metabolic shifts, KEGG pathway enrichment analysis was performed (Figure 7). The results revealed divergent metabolic shifts among the three morphotypes. In the LZ phenotype, significant enrichment was observed in linoleic acid metabolism and flavonoid biosynthesis. Since linoleic acid is a precursor to jasmonic acid (JA), a key stress hormone (He and Ding, 2020), this accumulation suggests that the LZ morphotype is in an active state of stress signaling and chemical defense mobilization. Conversely, the MZ phenotype showed predominant enrichment in steroid biosynthesis and fatty acid metabolism, supporting the hypothesis of physiological adjustment and membrane remodeling under chronic stress. The P phenotype highlighted phenylpropanoid biosynthesis and starch and sucrose metabolism, favoring the allocation of carbon sources towards structural polymers like lignin and cellulose.

Synthesizing these findings, we reconstructed a metabolic regulatory network (Figure 7D) to visualize the potential metabolic associations between the three morphotypes. Rather than acting as a dominant central hub, the phenylpropanoid pathway serves as a foundational, basal pathway from which downstream metabolic branches diverge based on the stress response strategy. Furthermore, an explicit distinction is observed between statistical significance and pathway impact (Figure 7). Flavonoid biosynthesis exhibits the highest statistical significance (-log10 p-value), indicating a coordinated, broad-scale accumulation of multiple defensive downstream metabolites. Conversely, linoleic acid (C18:2) metabolism shows lower statistical significance but the highest pathway impact score. To understand this discrepancy, it is essential to explicitly distinguish between linoleic acid and α-linolenic acid. While α-linolenic acid (C18:3) is the canonical, direct precursor for jasmonic acid (JA) biosynthesis via the octadecanoid pathway, linoleic acid serves as its vital upstream substrate. Linoleic acid must be desaturated to α-linolenic acid by fatty acid desaturases (FADs) to fuel JA synthesis. Thus, its exceptionally high pathway impact suggests that the mobilization and desaturation of the upstream linoleic acid pool act as a critical rate-limiting bottleneck; even minor metabolic fluxes at this node exert profound downstream effects on stress signaling. Under acute stress conditions (LZ model), the synthetic pathway is partitioned towards the synthesis of defensive flavonoids and sinapyl alcohol to form a dual chemical-physical barrier, likely driven by intense JA signaling initiated by this linoleic to α-linolenic acid cascade (Zhu et al., 2021). Under chronic adaptation (MZ model), the metabolic profile shifts towards lipids and steroids to maintain cellular integrity and systemic resin deposition. In the basic repair state (P model), the metabolic allocation is directed towards coniferaldehyde and lignin monomers to repair the hollow cork cambium physically.These distinct metabolic allocations support our proposed putative model of a “progressive metabolic continuum” (P → MZ → LZ), where the plant may dynamically adjusts its resource allocation from basic repair to chronic adaptation, and finally to an emergency defensive burst, Because these observations are based on cross-sectional metabolic profiling, this “progressive metabolic continuum” model remains a hypothesized framework representing metabolic gradients. It illustrates physiological associations and potential resource allocation strategies rather than proven causal successions.

### 3.5 Limitations of the study

Three limitations should be acknowledged. First, due to the scarcity of wild resources, LZ samples were collected from Laos, whereas P and MZ samples originated from Myanmar. While geographic factors may influence the metabolome, the observed metabolic profiles aligned strictly with their morphological functions (e.g., acute defense vs. structural repair), suggesting that physiological status is the dominant driver overriding geographic variations. Second, the proposed “progressive metabolic continuum” is a putative framework inferred from observed metabolic gradients and physiological functions. Because cross-sectional comparisons cannot directly capture real-time biological transitions, the P → MZ → LZ sequence remains a scientifically grounded hypothesis. Longitudinal monitoring of the resinification process in the same tree over an extended duration, ideally integrated with time-series transcriptomics or stable isotope tracing, would be required to definitively validate this dynamic biological progression. Third, due to the opportunistic and field-collected nature of these wild resources, precise metadata regarding the exact age of the mother trees, specific environmental microclimates, and the initial natural stress/infection triggers were unavailable. Consequently, while our multivariate models demonstrated robust metabolic clustering driven strictly by wood morphotype, we cannot entirely rule out the potential confounding influences of these unmeasured developmental and environmental variables. Future controlled induction experiments on cultivated trees of known age and standardized environmental conditions are necessary to decouple these factors.

## 4 Conclusion

In summary, this research successfully established a comprehensive and spatially resolved phytochemical map across distinct *Dracaena cochinchinensis* wood morphotypes. By leveraging an integrated workflow of UPLC-Q-TOF-MS/MS and Feature-Based Molecular Networking (FBMN), we achieved the systematic annotation of 299 specialized metabolites, effectively resolving the complex chemical space of this woody medicinal matrix. Our findings support a “progressive metabolic continuum” (P→MZ→LZ) model, illustrating a dynamic reprogramming of secondary metabolism driven by stress intensity. This continuum reflects a hierarchical resource allocation strategy: transitioning from lignin-mediated physical reinforcement in the P morphotype to steroid-based chronic adaptation in the MZ morphotype, and finally to an acute chemical defense burst of flavonoids and terpenoids in the LZ morphotype. These results provide a robust molecular framework for identifying high-value therapeutic compounds and offer critical theoretical guidance for the quality evaluation and sustainable development of *Dracaena cochinchinensis* as a prestigious woody medicinal resource.

## Data Availability

The dataset MSV000101733 has now been made publicly available in the MassIVE database.
