# Supplementary material for: Integrative metabolomics and molecular networking reveal the progressive metabolic continuum of resin formation in Dracaena cochinchinensis wood
Source: Front Mol Biosci. 2026 May 28;13:1795393. doi: 10.3389/fmolb.2026.1795393 (PMC13253446; doi:10.3389/fmolb.2026.1795393)
Supplement: Supplementary file 1 [file DataSheet1.docx]

Supplementary Material

**Supplementary Figure 1.** Total ion current chromatograms of resin-containing wood samples from different morphological types of *Dracaena cochinchinensis* (A: LZ, B: MZ, C: P).

**Supplementary Figure 2.** Feature-Based Molecular Network of resin-containing wood from *Dracaena cochinchinensis* in positive ion mode.

**Supplementary Figure 3.** Feature-Based Molecular network of resin-containing wood in *Dracaena cochinchinensis* in negative ion mode.

**Supplementary Figure 4.** Classification of non-volatile compounds from resin-containing wood of *Dracaena cochinchinensis*.

**Supplementary Figure 5.** Clustering of differential metabolite abundance between LZ group and MZ groups.

**Supplementary Figure 6.** Clustering of differential metabolite abundance between MZ group and P groups.

**Supplementary Figure 7.** Clustering of differential metabolite abundance between LZ group and P groups.

**Supplementary Figure 8.**  Heatmap integrating pairwise comparisons of differential compounds (P/LZ/MZ).

**Supplementary Table S1.** Specific information on non-volatile compounds annotated by UPLC-Q-TOF-MS/MS analysis in resin-containing wood from different morphological types of *Dracaena cochinchinensis*.

**Supplementary Table S2.** Parameters of OPLS-DA models.

**Supplementary Table S3.** Metabolites with significantly increased accumulation in the LZ group among differential compounds.

**Supplementary Table S4.** Metabolites with significantly increased accumulation in the MZ group among differential compounds.

**Supplementary Table S5.** Metabolites with significantly increased accumulation in the P group among differential compounds.


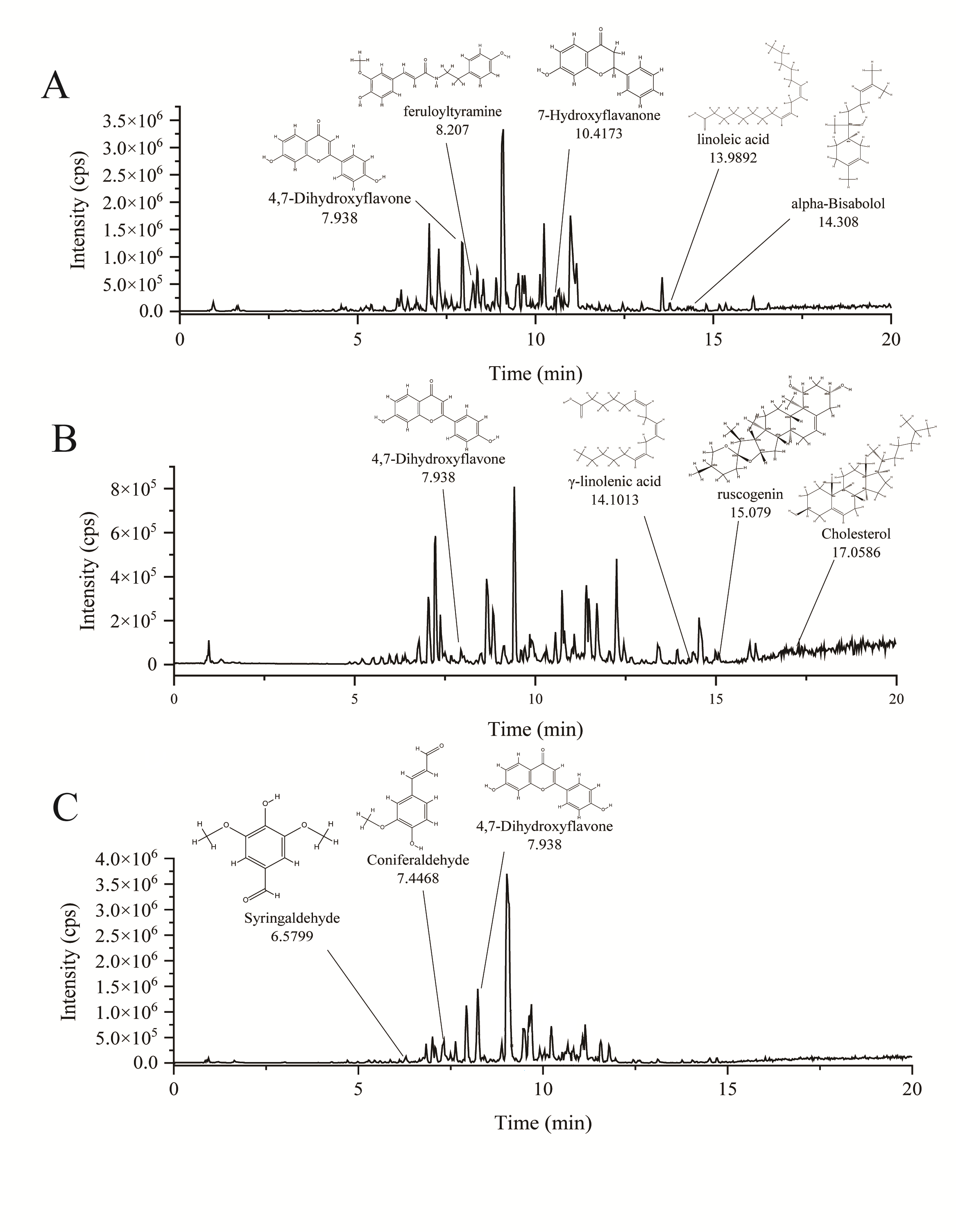


**Supplementary Figure 1.** Total ion current chromatograms of resin-containing wood samples from different morphological types of *Dracaena cochinchinensis* (A: LZ, B: MZ, C: P).


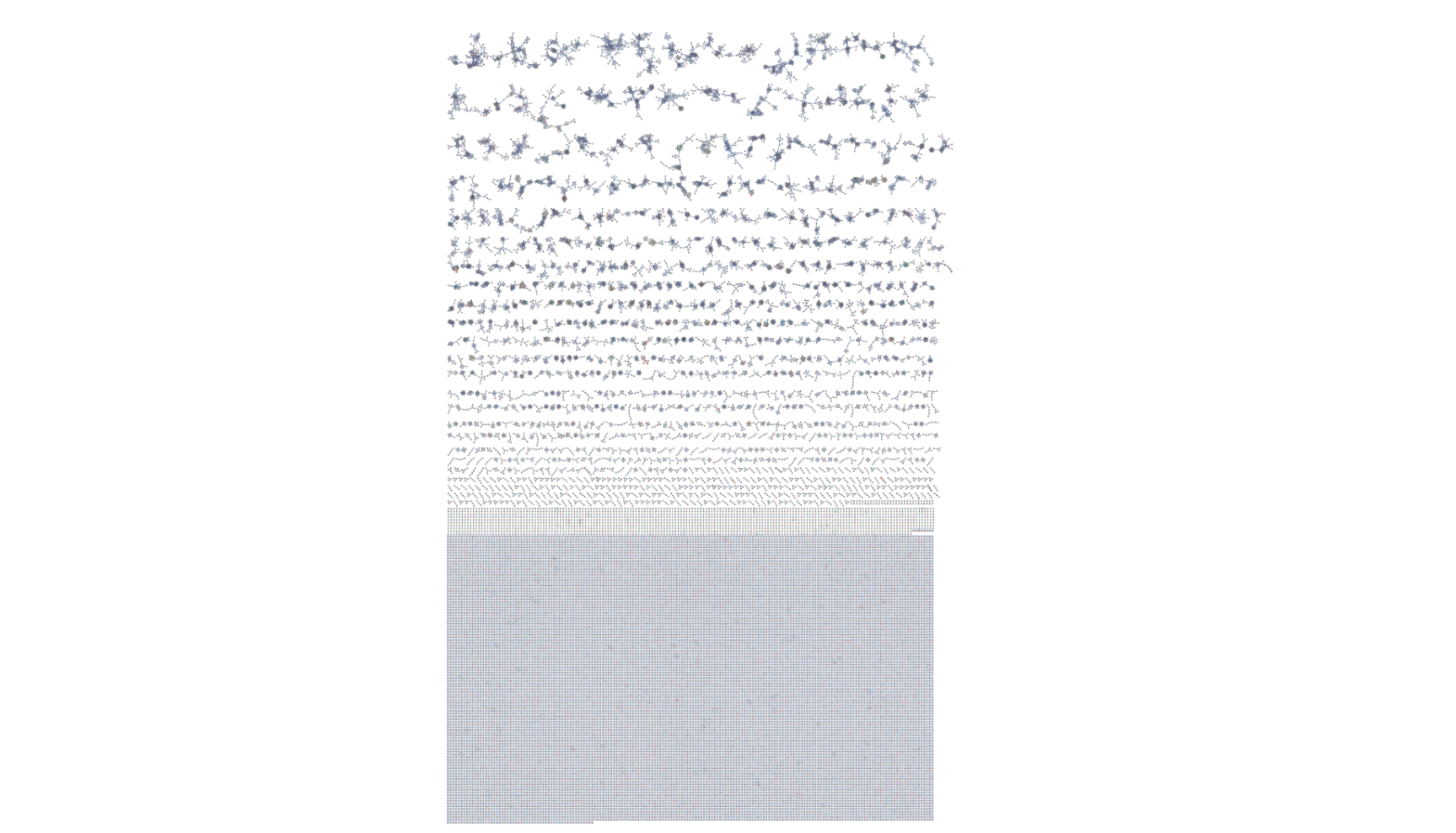


**Supplementary Figure 2.** Feature-Based Molecular Network of resin-containing wood from *Dracaena cochinchinensis* in positive ion mode.


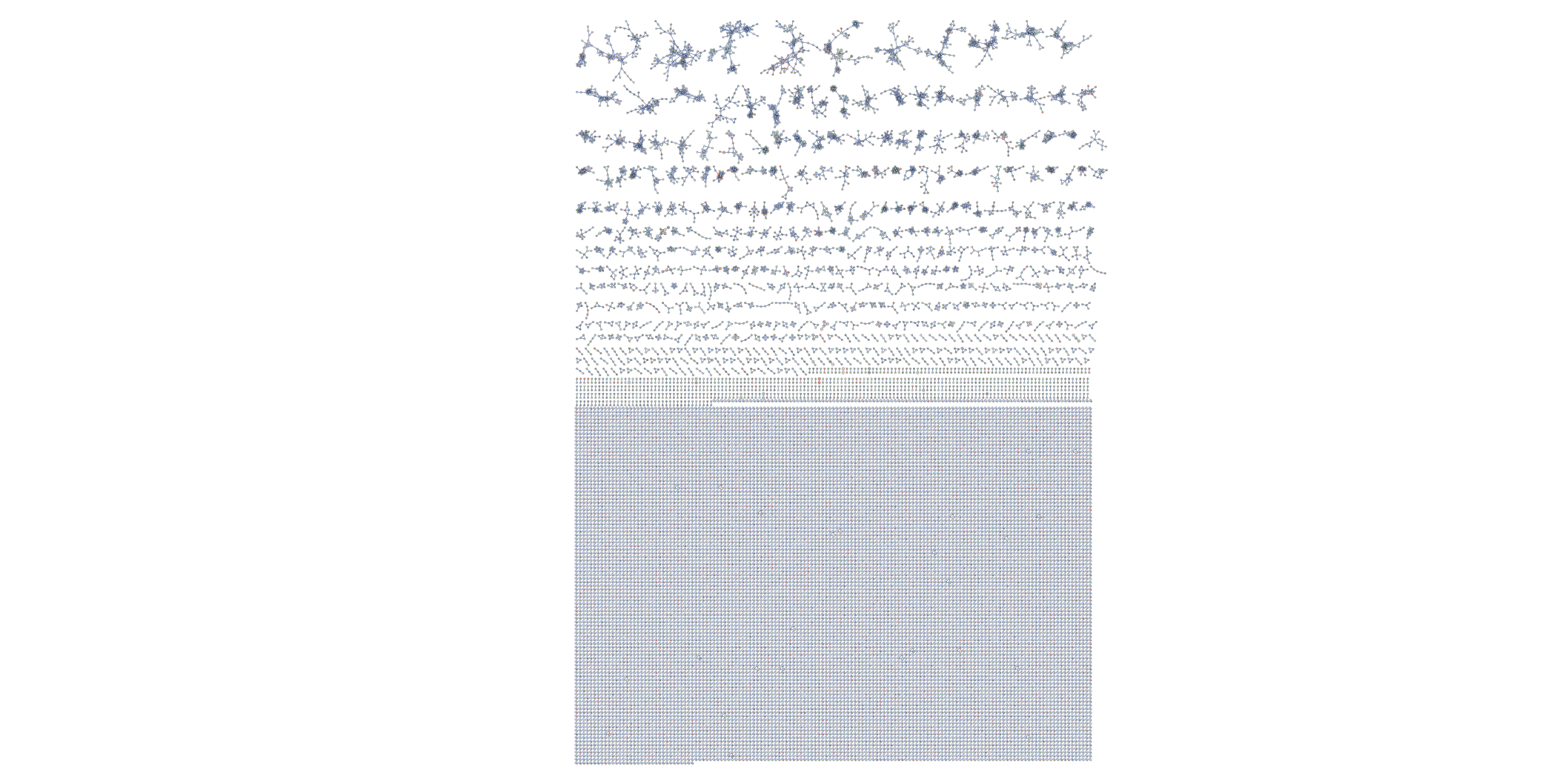


**Supplementary Figure 3.** Feature-Based Molecular network of resin-containing wood in *Dracaena cochinchinensis* in negative ion mode.


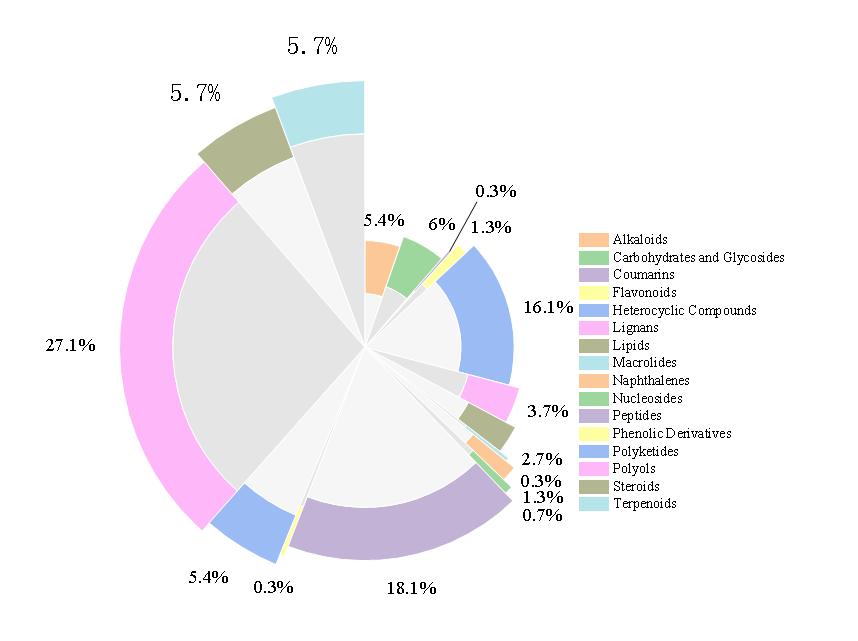


**Supplementary Figure 4.** Classification of non-volatile compounds from resin-containing wood of *Dracaena cochinchinensis*.


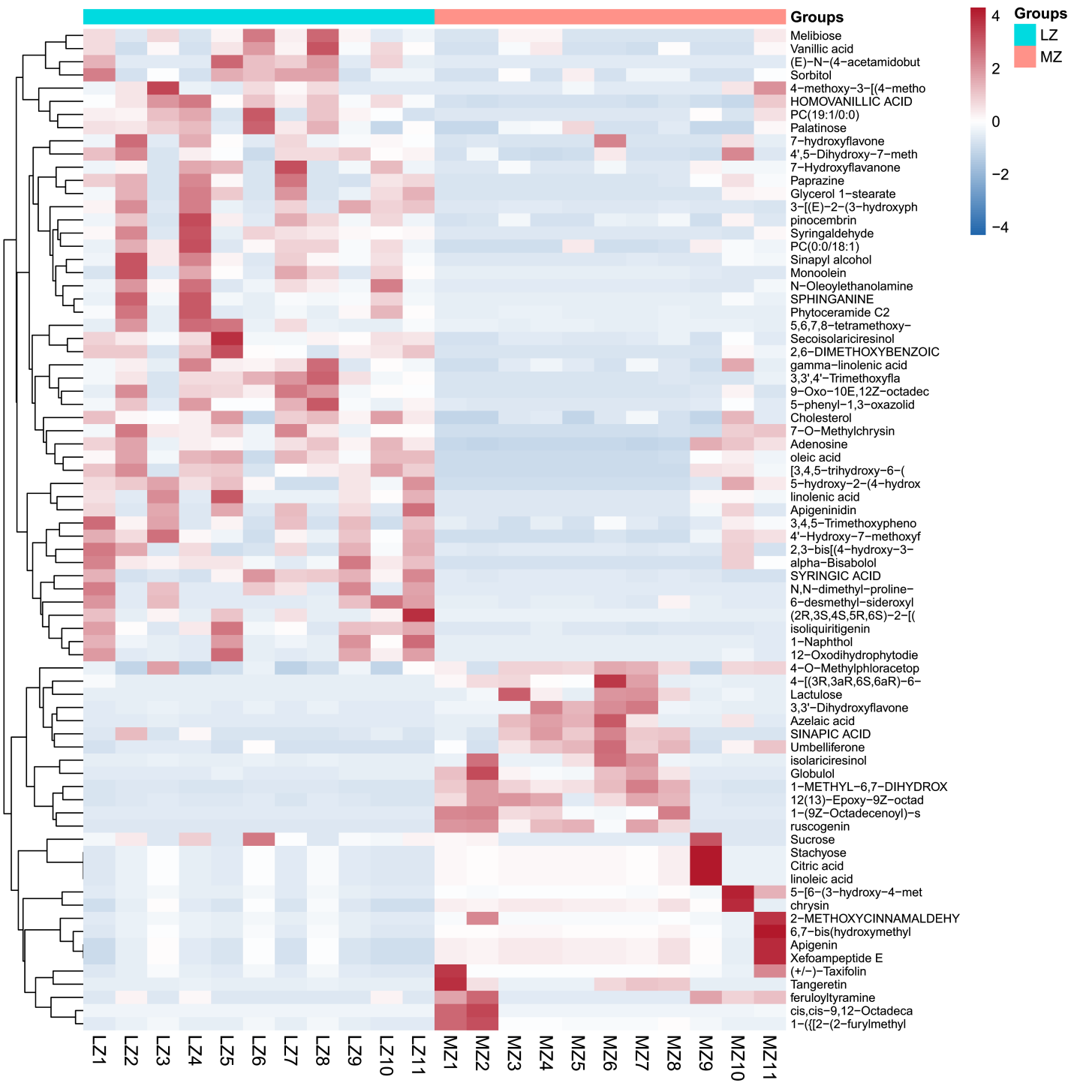


**Supplementary Figure 5.** Clustering of differential metabolite abundance between LZ group and MZ groups.


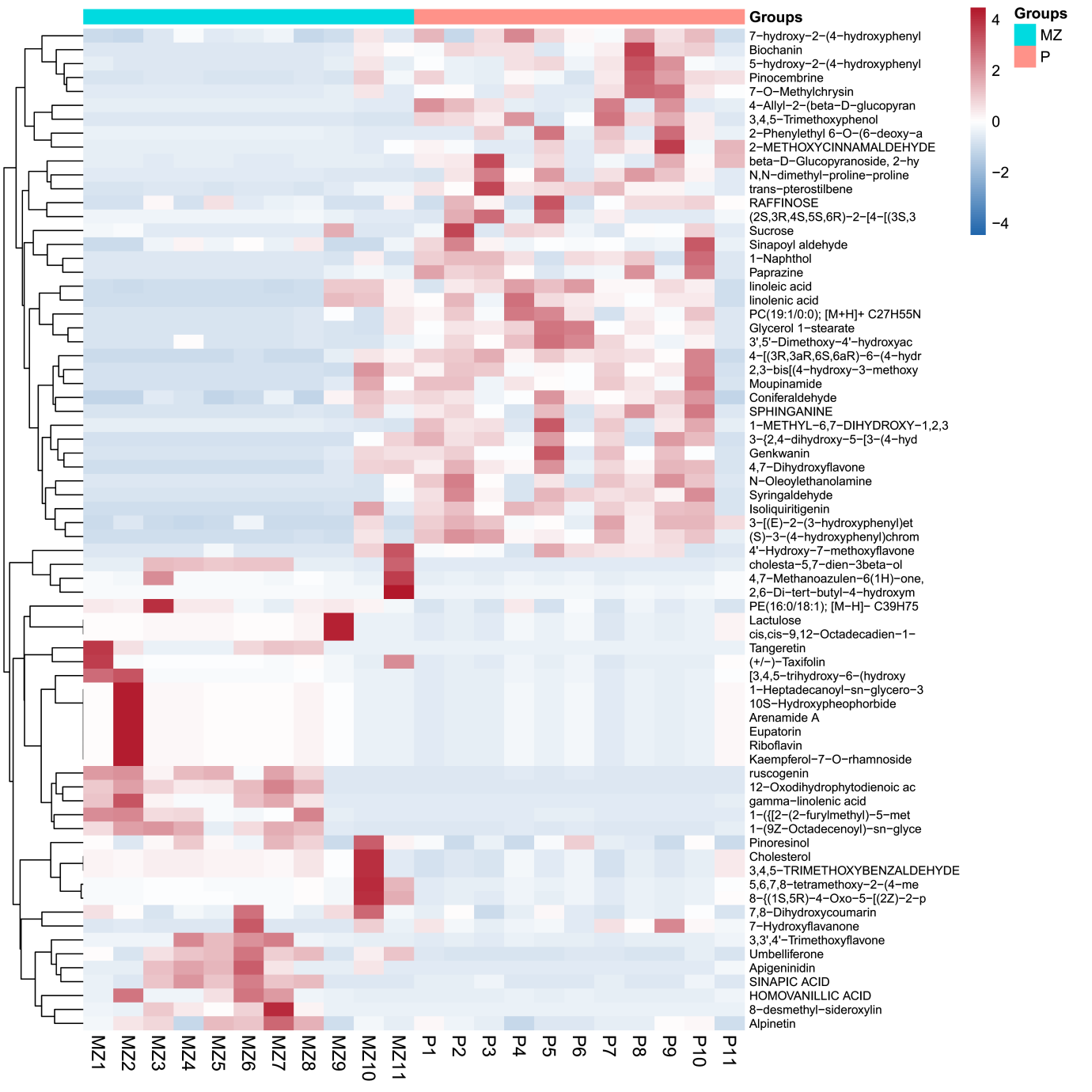


**Supplementary Figure 6.** Clustering of differential metabolite abundance between MZ group and P groups.


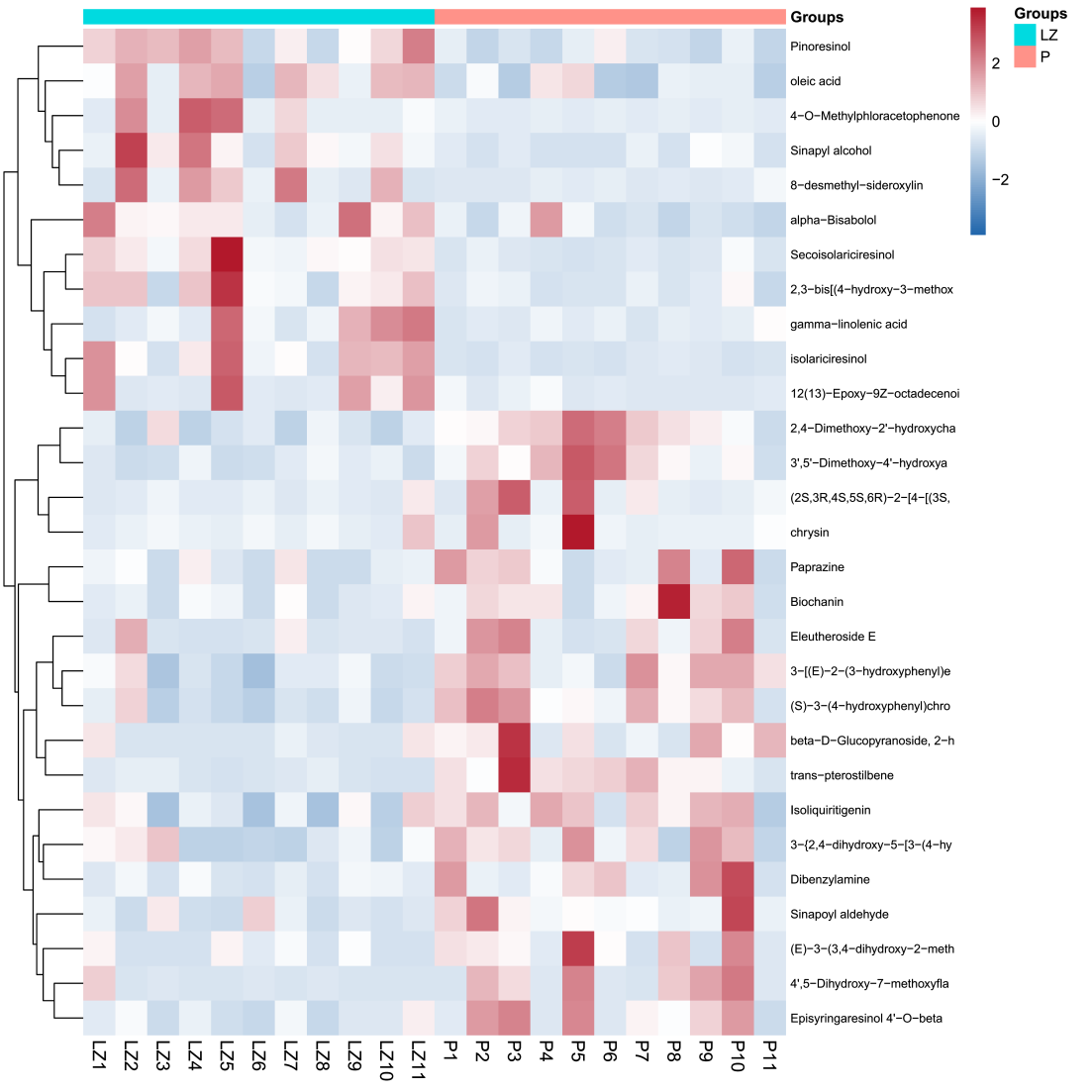


**Supplementary Figure 7.** Clustering of differential metabolite abundance between LZ group and P groups.


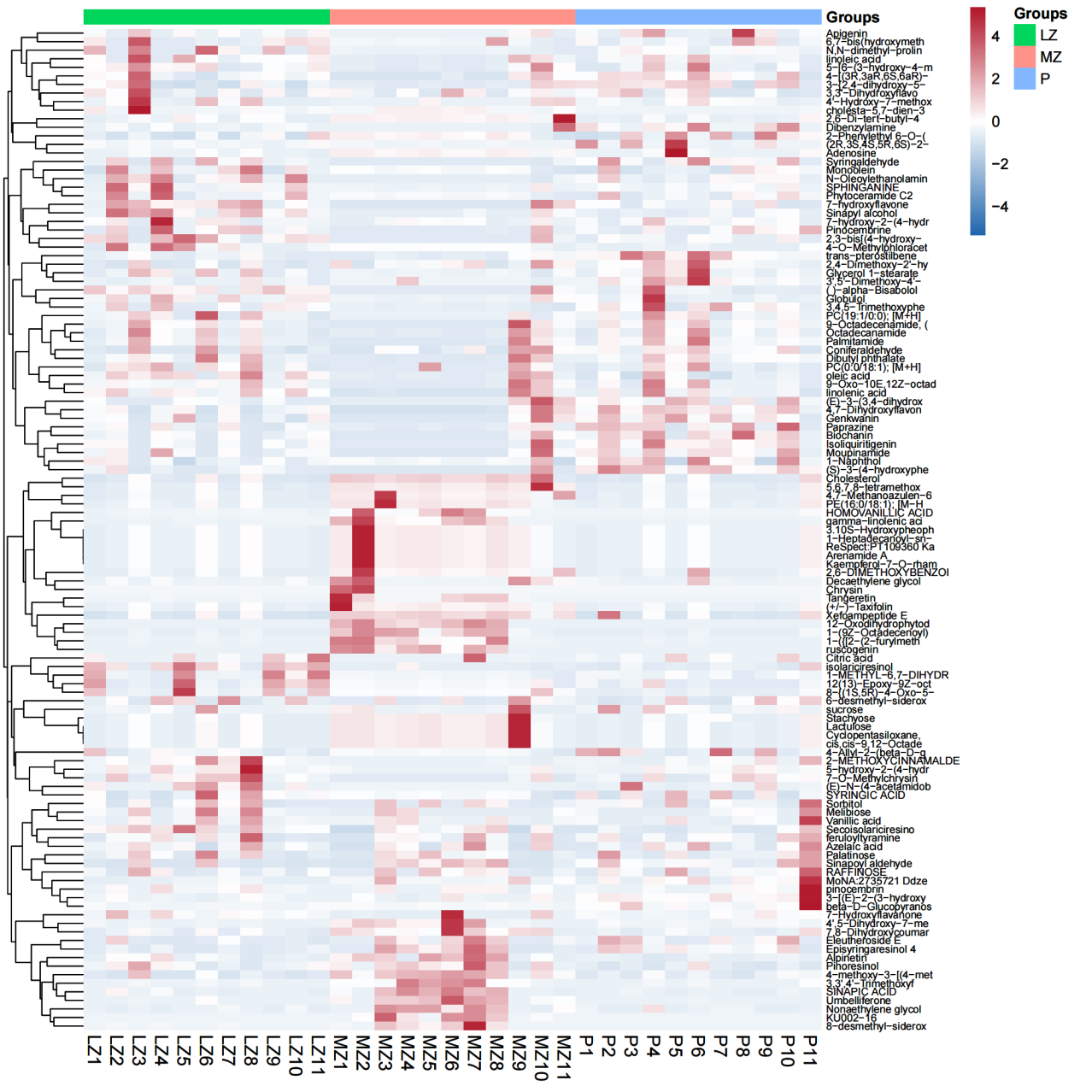


Supplementary Figure 8. Heatmap integrating pairwise comparisons of differential compounds (P/LZ/MZ).

**Supplementary Table S1.** Specific information on non-volatile compounds annotated by UPLC-Q-TOF-MS/MS analysis in resin-containing wood from different morphological types of *Dracaena cochinchinensis*.

| NO. | Compound name | Formula | Adduct | RT/min | Theoretical *m/z* | Measured *m/z* | Error ppm | MS/MS Fragments | IonMode | MSI Level | Form |
| --- | --- | --- | --- | --- | --- | --- | --- | --- | --- | --- | --- |
| Terpenoids | | | | | | | | | | | |
| 1 | methyl (1S,5S,9S,10R,15R)-15-acetyloxy-6-hydroxy-4,5,7,10,14,14-hexamethyl-8,18-dioxo-19-oxapentacyclo[10.5.2.01,13.02,10.05,9]nonadeca-3,6-diene-9-carboxylate | C_28_H_36_O_8_ | [M-H]^-^ | 11.1787 | 499.2337 | 499.2346 | 1.8 | 59.0124, 423.2175, 467.2073, 439.2122 | Negative | 2 | LZ |
| 2 | Longicamphenylone | C_14_H_22_O | [M+H]^+^ | 13.8281 | 207.1744 | 207.1744 | 0 | 123.0800, 95.0854 105.0699, 93.0706, 67.0559 | Positive | 2 | LZ、MZ |
| 3 | Hulupinic acid | C_15_H_20_O_4_ | [M+H]^+^ | 13.6645 | 265.1434 | 265.1438 | 1.51 | 149.0595, 121.0647, 93.0697, 177.0545, 163.0752 | Positive | 2 | LZ、P |
| 4 | Globulol | C_15_H_26_O | [M+H-H2O]^+^ | 14.5314 | 205.1951 | 205.1951 | 0 | 107.0857, 109.1012, 93.0700, 95.0858, 67.0546 | Positive | 2 | LZ、MZ、P |
| 5 | Dihydroactinidiolide | C_11_H_16_O_2_ | [M+H]^+^ | 7.0138 | 181.1223 | 181.1223 | 0 | 91.0544, 77.0397, 79.0552, 93.0699, 105.0702 | Positive | 2 | LZ |
| 6 | Canangalia H | C_15_H_26_O_2_ | [M+H-H2O]^+^ | 14.1277 | 221.1901 | 249.1854 | 0 | 189.1638, 119.0856, 133.1013, 217.1587, 147.1170,93.0703, 105.0702, 95.0859, 109.1014, 135.1169 | Positive | 2 | LZ |
| 7 | alpha-Bisabolol | C_15_H_26_O | [M+H-H2O]^+^ | 14.3081 | 205.1951 | 205.1951 | 0 | 55.0540, 69.0695, 81.0695, 95.0851, 121.1009, 205.1951 | Positive | 2 | LZ、MZ、P |
| 8 | abscisic acid | C_15_H_20_O_4_ | [M-H]^-^ | 8.516 | 263.1290 | 263.1296 | 2.28 | 219.1388, 204.1154, 203.1077, 152.0840, 151.0762 | Negative | 2 | LZ |
| 9 | 7b,9-Dihydroxy-3-(hydroxymethyl)-1,1,6,8-tetramethyl-5-oxo-1,1a,1b,4,4a,5,7a,7b,8,9-decahydro-9aH-cyclopropa[3,4]benzo[1,2-e]azulen-9a-yl acetate | C_22_H_30_O_6_ | [M+ACN+H]^+^ | 11.9625 | 432.2381 | 432.2374 | -1.62 | 119.0859, 107.0853, 135.0812, 105.0694, 91.0537 | Positive | 2 | LZ、MZ、P |
| 10 | 4,7-Methanoazulen-6(1H)-one, 2,4,5,7,8,8a-hexahydro-7-hydroxy-9-(hydroxymethyl)-1,4,9-trimethyl-, (1S,4R,7R,8aS)- | C_15_H_24_O_3_ | [M+H-H2O]^+^ | 8.4603 | 233.1536 | 233.1539 | 1.29 | 91.0541, 105.0693, 147.0809, 119.0852, 105.0445 | Positive | 2 | LZ |
| 11 | 2(1H)-Naphthalenone, 4a,5,6,7,8,8a-hexahydro-6-hydroxy-3,8-dimethyl-5-(1-methylethyl)-, (4aR,5S,6S,8R,8aS)- | C_15_H_26_O_2_ | [M+H-H2O]^+^ | 14.7952 | 219.1744 | 219.1743 | -0.46 | 121.0656, 105.0702, 91.0543, 145.1019, 107.0855, | Positive | 2 | LZ、MZ、P |
| 12 | 1-methyl-4-methylidene-7-(propan-2-yl)-1,2,3,3a,4,5,6,8a-octahydroazulen-1-ol | C_15_H_24_O | [M+H-H2O]^+^ | 14.0988 | 203.1794 | 203.1794 | 0 | 147.1166, 105.0698, 95.0856, 119.0854, 109.1011 | Positive | 2 | LZ、MZ、P |
| 13 | 1H-3a,6-Methanoazulene-3-carboxylic acid, octahydro-7,7-dimethyl-8-methylene-, (3S,3aR,6R,8aS)- | C_14_H_22_O_2_ | [M+H]^+^ | 14.4175 | 223.1693 | 235.1693 | 0 | 105.0699, 189.1646, 133.1014, 119.0855, 91.0546 | Positive | 2 | MZ |
| 14 | 1,8-Azulenediol, 1,2,3,3a,4,7,8,8a-octahydro-3a,6-dimethyl-1-(1-methylethyl)-, (1R,3aR,8S,8aS)- | C_15_H_26_O_2_ | [M+H-H2O]^+^ | 14.292 | 221.1901 | 221.1901 | 0 | 119.0857, 105.0701, 95.0858, 147.1169,147.1169 | Positive | 2 | LZ、MZ |
| 15 | (3E)-7-Hydroxy-3,7-dimethyl-3-octen-1-yl 6-O-(6-deoxy-alpha-L-mannopyranosyl)-beta-D-glucopyranoside | C_22_H_40_O_11_ | [M+FA-H]^-^ | 6.5185 | 525.2552 | 525.2557 | 0.95 | 163.0615, 479.2476, 205.0732, 265.0952, 143.0371 | Negative | 2 | LZ、MZ、P |
| 16 | (1S,8R,9R)-8-hydroxy-4-(propan-2-ylidene)-10-oxatricyclo[7.2.1.01,5]dodecane-8-carboxylic acid | C_15_H_22_O_4_ | [M-H]^-^ | 10.3445 | 265.1445 | 265.1449 | 1.51 | 219.1386, 189.1278, 57.0332, 220.1419 | Negative | 2 | LZ、P |
| Steroids | | | | | | | | | | | |
| 17 | Spirostane -2H, + 1O, O-Pen-dHex | C_38_H_60_O_13_ | [M+HCOO]^-^ | 12.0256 | 753.4067 | 753.4086 | 2.52 | 707.3995, 708.4023, 709.4276, 131.0307, 709.4006 | Negative | 2 | LZ、MZ、P |
| 18 | Spirostane + 1O, -2H, O-Hex-dHex-dHex | C_45_H_72_O_18_ | [M+HCOO]^-^ | 12.6478 | 929.4751 | 929.4774 | 2.47 | 883.4674, 884.4794, 885.4920, 737.4220, 922.3046 | Negative | 2 | LZ、MZ、P |
| 19 | Spirost-5-en-3-ol, (3beta,25R)- | C_27_H_42_O_3_ | [M+H-H2O]^+^ | 12.6225 | 397.3094 | 397.3096 | 0.5 | 253.1955, 147.1175, 397.3105, 145.1015,105.070 | Positive | 2 | LZ、MZ、P |
| 20 | ruscogenin | C_27_H_42_O_4_ | [M+H]^+^ | 15.079 | 431.3156 | 431.3148 | -1.85 | 431.3240, 139.07, 251.18, 269.19, 287.20, | Positive | 2 | MZ、P |
| 21 | Furostane base -2H + O-Hex, O-Hex-dHex-dHex | C_51_H_82_O_23_ | [M+HCOO]^-^ | 8.7345 | 1093.5436 | 1093.5448 | 1.1 | 1047.5397, 1048.5443, 1049.5372, 1050.5664, 1046.4819 | Negative | 2 | LZ、MZ、P |
| 22 | Furostane base -2H + 1O, O-Hex, O-Pen-dHex | C_44_H_72_O_17_ | [M+H-H2O]^+^ | 7.3664 | 871.4686 | 871.4611 | -8.61 | 269.1910, 287.2030, 709.4134, 251.1756,147.0643 | Positive | 2 | LZ、MZ、P |
| 23 | diosgenin | C_27_H_42_O_3_ | [M+H]^+^ | 12.6096 | 415.3207 | 415.32 | -1.69 | 271.2056, 253.1948, 159.1169, 157.1011,147.1171 | Positive | 2 | LZ、MZ、P |
| 24 | Dioscin | C_45_H_72_O_16_ | [M+H]^+^ | 12.5998 | 869.4893 | 869.4868 | -2.88 | 253.1953, 129.0544, 147.0652, 271.2059,239.0919 | Positive | 2 | LZ、MZ、P |
| 25 | Cholesterol | C_27_H_46_O | [M+H-H2O]^+^ | 17.0586 | 369.3516 | 369.3513 | -0.81 | 147.1165, 161.1321, 135.1170, 149.1329, 175.1485 | Positive | 2 | MZ |
| 26 | cholesta-5,7-dien-3beta-ol | C_27_H_44_O | [M+H]^+^ | 16.3387 | 385.3465 | 385.3457 | -2.08 | 159.1167, 161.1325, 145.1009, 147.1169, 147.1169, | Positive | 2 | LZ、MZ |
| 27 | Cholecalciferol | C_27_H_44_O | [M+H]^+^ | 19.8348 | 385.3465 | 385.3459 | -1.56 | 97.0644, 109.0644, 385.3481, 95.0852, 95.0852 | Positive | 2 | MZ |
| 28 | Boldione | C_19_H_24_O_2_ | [M+H]^+^ | 11.046 | 285.1849 | 285.185 | 0.35 | 121.0650, 151.1111, 147.1165, 267.1725, 107.0860 | Positive | 2 | MZ |
| 29 | (3β,22α,25R)-26-(β-D-glucopyranosyloxy)-22-hydroxyfurost-5-en-3-yl O-α-L-rhamnopyranosyl-(1→2)-O-[α-L-rhamnopyranosyl-(1→4)]-β-D-glucopyranoside | C_51_H_84_O_22_ | [M+H-H2O]^+^ | 8.7415 | 1031.5319 | 1031.539 | 6.88 | 869.491516, 870.495911, 725.380066, 727.391785, 723.437439 | Positive | 2 | LZ、MZ、P |
| 30 | 7-ketocholesterol | C_27_H_44_O_2_ | [M+H]^+^ | 19.398 | 401.3414 | 401.3404 | -2.49 | 383.330505, 365.320099, 175.111893, 159.117004, 95.085503 | Positive | 2 | LZ、MZ、P |
| 31 | (3S,8S,9S,10R,13R,14S,17R)-17-((2R)-7-hydroxy-6-methylheptan-2-yl)-10,13-dimethyl-2,3,4,7,8,9,10,11,12,13,14,15,16,17-tetradecahydro-1H-cyclopenta[a]phenanthren-3-ol | C_24_H_42_O_2_ | [M+H-2H2O]^+^ | 16.3291 | 367.3360 | 367.3353 | -1.91 | 213.1630, 199.1470, 187.1476, 173.1316, 161.1316 | Positive | 2 | LZ、MZ、P |
| 32 | (3beta,22R,25R)-26-(beta-D-Glucopyranosyloxy)-22-hydroxyfurost-5-en-3-yl 6-deoxy-alpha-L-mannopyranosyl-(1->2)-[beta-D-glucopyranosyl-(1->4)]-beta-D-glucopyranoside | C_51_H_84_O_23_ | [M+H-H2O]^+^ | 8.8633 | 1047.5268 | 1047.533 | 5.92 | 415.3217, 271.2060, 253.1961, 129.0555, 85.0273 | Positive | 2 | LZ、MZ、P |
| 33 | (1beta,3beta,9xi,14xi)-3-Hydroxyspirosta-5,25(27)-dien-1-yl 2-O-(6-deoxy-alpha-L-mannopyranosyl)-alpha-L-arabinopyranoside | C_38_H_58_O_12_ | [M+H]^+^ | 11.772 | 707.3998 | 707.3983 | -2.12 | 411.2921, 393.2795, 287.2016, 269.1909, 251.1803 | Positive | 2 | LZ、MZ、P |
| 34 | (1beta,3alpha,9xi,14xi)-1-Hydroxyspirosta-5,25(27)-dien-3-yl beta-D-glucopyranosyl-(1->3)-6-deoxy-alpha-L-mannopyranosyl-(1->2)-(3xi)-alpha-D-threo-pentopyranoside | C_44_H_68_O_17_ | [M+H]^+^ | 7.3257 | 869.4529 | 869.4506 | -2.65 | 411.2910, 393.2802, 287.2008, 269.1910, 251.1800 | Positive | 2 | LZ、MZ、P |
| Polyols | | | | | | | | | | | |
| 35 | AC1L1X1Z | C_23_H_46_N_6_O_13_ | [M+Na]^+^ | 16.7291 | 637.2619 | 637.3032 | 64.9 | 581.2426, 393.0858, 337.0233, 147.1169, 57.0704 | Positive | 2 | LZ、MZ、P |
| Polyketides | | | | | | | | | | | |
| 36 | Fallacinol | C_16_H_12_O_6_ | [M-H]^-^ | 6.4948 | 299.0561 | 299.0567 | 2.01 | 256.0420, 255.0334, 211.0425, 239.0355, 227.0387 | Negative | 2 | MZ、P |
| 37 | Emodin | C_15_H_10_O_5_ | [M-H]^-^ | 12.1982 | 269.0455 | 269.0462 | 2.6 | 225.0553, 270.0485, 241.0500, 226.0584, 182.0408 | Negative | 2 | LZ、MZ |
| 38 | Duclauxin | C_29_H_22_O_11_ | [M+H]^+^ | 11.3792 | 547.1235 | 547.1261 | 4.75 | N/A | Positive | 2 | LZ |
| 39 | 1-({[2-(2-furylmethyl)-5-methylpyrrolidinyl]amino}methylene)-7-[8-({[2-(2-fury lmethyl)pyrrolidinyl]amino}methylene)-1,6-dihydroxy-3-methyl-5-(methylethyl)-7 -oxo(2-naphthyl)]-3,8-dihydroxy-6-methyl-4-(methylethyl)naphthalen-2-one | C_24_H_30_O_6_ | [M+2H]^+^ | 12.2917 | 416.2193 | 415.2107 | -20.6 | 119.0855, 91.0543, 117.0697, 133.0648, 107.0854 | Positive | 2 | LZ、MZ、P |
| Phenolic Derivatives | | | | | | | | | | | |
| 40 | Vanillic acid | C_8_H_8_O_4_ | [M-H]^-^ | 6.4059 | 167.0350 | 167.0357 | 4.19 | 152.0120, 108.0220, 123.0450, 91.0189, 123.0100 | Negative | 2 | LZ、MZ、P |
| 41 | Undulatoside A | C_16_H_22_O_9_ | [M+H]^+^ | 5.7408 | 359.1337 | 355.1027 | -11.4 | 193.0494, 151.0384, 153.0176, 67.0190, 147.0437 | Positive | 2 | LZ、P |
| 42 | trans-resveratrol | C_14_H_12_O_3_ | [M-H]^-^ | 7.809 | 227.0714 | 227.0716 | 0.88 | 143.0497, 185.0602, 183.0807, 182.0730, 181.0653 | Negative | 2 | LZ、P |
| 43 | trans-pterostilbene | C_16_H_16_O_3_ | [M+H]^+^ | 11.5723 | 257.1172 | 257.1172 | 0 | 256.1085, 242.0931, 225.0928, 133.0652, 105.0694 | Positive | 2 | LZ、MZ、P |
| 44 | trans-Ferulic acid | C_10_H_10_O_4_ | [M+H-H2O]^+^ | 7.4406 | 177.0546 | 177.0545 | -0.56 | 89.0392, 117.0339, 149.0633, 134.0362, 145.0267 | Positive | 2 | LZ、MZ、P |
| 45 | Syringic acid | C_9_H_10_O_5_ | [M+H]^+^ | 5.6498 | 199.0601 | 199.0601 | 0 | 140.0468, 95.0491, 125.0233, 97.0285, 53.0385 | Positive | 2 | LZ、P |
| 46 | Syringaldehyde | C_9_H_10_O_4_ | [M+H]^+^ | 6.5799 | 183.0652 | 183.0652 | 0 | 140.0520, 123.0470, 95.0540, 77.0410, 67.0560 | Positive | 2 | LZ、MZ、P |
| 47 | Sinapyl alcohol | C_11_H_14_O_4_ | [M+H-H2O]^+^ | 10.2274 | 193.0860 | 193.0859 | -0.52 | 161.0598, 105.0699, 133.0649, 115.0542, 103.0542 | Positive | 2 | LZ、MZ、P |
| 48 | Sinapoyl aldehyde | C_11_H_12_O_4_ | [M-H]^-^ | 7.4248 | 207.0663 | 207.0670 | 3.38 | 149.0231, 177.0222, 121.0289, 93.0304, 77.0384 | Negative | 2 | LZ、MZ、P |
| 49 | Sinapic acid | C_11_H_12_O_5_ | [M+H-H2O]^+^ | 7.8177 | 207.0652 | 207.0652 | 0 | 91.0544, 175.0389, 119.0491, 147.0440, 192.0415 | Positive | 2 | LZ、MZ、P |
| 50 | Paprazine | C_17_H_17_NO_3_ | [M+H]^+^ | 7.9615 | 284.1281 | 284.1288 | 2.46 | 284.0630, 164.0710, 147.0440, 121.0650, 119.0490 | Positive | 2 | LZ、MZ、P |
| 51 | Myristicin | C_11_H_12_O_3_ | [M+H]^+^ | 12.8992 | 193.0860 | 193.0855 | -2.59 | 133.0646, 105.0694, 115.0536, 118.0373, 105.0430 | Positive | 2 | LZ、MZ |
| 52 | Moupinamide | C_18_H_19_NO_4_ | [M+H]^+^ | 8.2444 | 314.1387 | 314.1388 | 0.32 | 177.0540, 145.0280, 121.0650, 117.0330, 103.0540 | Positive | 2 | LZ、MZ、P |
| 53 | Methyl vanillate | C_9_H_10_O_4_ | [M+H]^+^ | 8.4613 | 183.0652 | 183.0650 | -1.09 | 151.0390, 124.0519, 107.0491, 79.0542, 77.0386 | Positive | 2 | LZ、MZ |
| 54 | methyl 4-hydroxy-3,5-dimethoxybenzoate | C_10_H_12_O_5_ | [M+H]^+^ | 7.9389 | 213.0758 | 213.0758 | 0 | 181.0560, 154.0680, 139.0460, 120.0250, 93.0370 | Positive | 2 | LZ、P |
| 55 | Homovanillic acid | C_9_H_10_O_4_ | [M+H]^+^ | 5.6211 | 183.0652 | 183.0652 | 0 | 122.0295, 94.0341, 77.0328, 66.0415, 65.0323 | Positive | 2 | MZ |
| 56 | Gnemontanin G | C_28_H_22_O_7_ | [M+H]^+^ | 8.4859 | 471.1438 | 471.1436 | -0.42 | 107.0494, 215.0699, 365.1013, 255.0647, 123.0442 | Positive | 2 | P |
| 57 | feruloyltyramine | C_18_H_19_NO_4_ | [M-H]^-^ | 8.207 | 312.1241 | 312.1250 | 2.88 | 148.0521, 135.0441, 190.0503, 178.0503, 147.0441 | Negative | 2 | LZ、MZ、P |
| 58 | Ferulic acid | C_10_H_10_O_4_ | [M-H]^-^ | 6.734 | 193.0506 | 193.0514 | 4.14 | 134.0375, 133.0302, 178.0281, 149.0610, 132.0228 | Negative | 2 | LZ、MZ、P |
| 59 | Ferulate | C_10_H_10_O_4_ | [M+H]^+^ | 7.28 | 195.0652 | 195.0647 | -2.56 | 177.0532, 145.0284, 117.0327, 89.0396, 63.0247 | Positive | 2 | MZ |
| 60 | Dihydro-ferulic acid | C_10_H_12_O_4_ | [M-H]^-^ | 6.5036 | 195.0663 | 195.0664 | 0.51 | 121.0293, 135.0447, 93.0350, 136.0523, 122.0321 | Negative | 2 | LZ、P |
| 61 | Coniferaldehyde | C_10_H_10_O_3_ | [M+H]^+^ | 7.4468 | 179.0703 | 179.0701 | -1.12 | 146.0363, 136.0519, 119.0491, 118.0413, 91.0542 | Positive | 2 | LZ、MZ、P |
| 62 | cis-resveratrol | C_14_H_12_O_3_ | [M+H]^+^ | 7.8428 | 229.0860 | 229.0865 | 2.18 | 181.0658, 165.0708, 153.0696, 107.0486, 91.0541 | Positive | 2 | P |
| 63 | beta-D-Glucopyranoside, 2-hydroxy-4-(2-propen-1-yl)phenyl 6-O-beta-D-glucopyranosyl- | C_21_H_30_O_12_ | [M-H]^-^ | 7.1235 | 473.1665 | 473.1665 | 0 | 149.0605, 323.0985, 148.0545, 179.0553, 161.0449 | Negative | 2 | LZ、P |
| 64 | 5-[(E)-2-(4-hydroxy-3-methoxyphenyl)ethenyl]benzene-1,3-diol | C_15_H_14_O_4_ | [M+H]^+^ | 10.7299 | 259.0965 | 259.0954 | -4.24 | 181.0640, 153.0708, 152.0614, 199.0754, 135.0423 | Positive | 2 | LZ |
| 65 | 4-O-Methylphloracetophenone | C_9_H_10_O_4_ | [M-H]^-^ | 9.0967 | 181.0506 | 181.0510 | 2.21 | 166.0262, 138.0311, 95.0125, 83.0125, 124.0154 | Negative | 2 | LZ、P |
| 66 | 4-methoxy-3-[(4-methoxyphenoxy)methyl]benzaldehyde | C_16_H_16_O_4_ | [M+H]^+^ | 10.8449 | 273.1121 | 273.1121 | 0 | 149.0596, 91.0544, 137.0597, 119.0491, 93.0700 | Positive | 2 | LZ、MZ、P |
| 67 | 4-Allyl-2-(beta-D-glucopyranosyloxy)phenyl beta-D-glucopyranoside | C_21_H_30_O_12_ | [M+Na]^+^ | 5.951 | 497.1629 | 497.1621 | -1.61 | 335.1113, , 171.0405, 336.1145, 185.0440 | Positive | 2 | LZ、P |
| 68 | 3-Hydroxy-4-methoxycinnamic acid | C_10_H_10_O_4_ | [M+H-H2O]^+^ | 8.8867 | 177.0546 | 177.0546 | 0 | 89.0393, 117.0327, 134.0370, 149.0585, 145.0287 | Positive | 2 | LZ、MZ、P |
| 69 | 3-[(E)-2-(3-hydroxyphenyl)ethenyl]-5-methoxyphenol | C_15_H_14_O_3_ | [M-H]^-^ | 9.9526 | 241.0870 | 241.0876 | 2.49 | 225.0554, 197.0602, 181.0652, 183.0444, 226.0629 | Negative | 2 | LZ、MZ、P |
| 70 | 3',5'-Dimethoxy-4'-hydroxyacetophenone | C_10_H_12_O_4_ | [M+H]^+^ | 6.9214 | 197.0808 | 197.0808 | 0 | 181.0513, 156.0750, 155.0698, 140.0479, 140.0338 | Positive | 2 | LZ、MZ、P |
| 71 | 3,4,5-Trimethoxyphenol | C_9_H_12_O_4_ | [M+H]^+^ | 6.6202 | 185.0808 | 185.0807 | -0.54 | 154.0624, 153.0546, 139.0390, 125.0597, 110.0362 | Positive | 2 | LZ、P |
| 72 | 3,4,5-Trimethoxycinnamic acid | C_12_H_14_O_5_ | [M+H-H2O]^+^ | 7.1162 | 221.0808 | 221.0812 | 1.81 | 190.0620, 206.0568, 193.0869, 163.0386, 147.0442 | Positive | 2 | LZ、MZ、P |
| 73 | 2-Phenylethyl 6-O-(6-deoxy-alpha-L-mannopyranosyl)-beta-D-glucopyranoside | C_20_H_30_O_10_ | [M+NH4]^+^ | 6.2839 | 448.2177 | 448.2172 | -1.12 | 147.0653, 145.0495, 129.0538, 85.0277, 83.0481 | Positive | 2 | LZ、P |
| 74 | 2-Methoxycinnamaldehyde | C_10_H_10_O_2_ | [M+H]^+^ | 10.7157 | 163.0753 | 163.0752 | -0.61 | 91.0553, 79.0547, 77.0393, 65.0395, 51.0232 | Positive | 2 | LZ、MZ、P |
| 75 | 2,8-Dimethyl-5,7-dimethoxychromone | C_13_H_14_O_4_ | [M+H]^+^ | 9.8052 | 235.0965 | 235.0966 | 0.43 | 220.0747, 205.0513, 189.0560, 219.0666, 191.0716 | Positive | 2 | P |
| 76 | 2,6-Di-tert-butyl-4-hydroxymethylphenol | C_15_H_24_O_2_ | [M+H-H2O]^+^ | 12.5264 | 219.1744 | 219.1744 | 0 | 203.1426, 133.1014, 121.0993, 119.0844, 91.0543 | Positive | 2 | LZ、MZ |
| 77 | 2,6-Dimethoxybenzoic acid | C_9_H_10_O_4_ | [M+H]^+^ | 7.5446 | 183.0652 | 183.0655 | 1.64 | 107.0124, 77.0378, 122.0359, 92.0254, 79.0174 | Positive | 2 | MZ、P |
| 78 | 2,3-Dihydroxybenzoic acid | C_7_H_6_O_4_ | [M-H]^-^ | 4.2561 | 153.0193 | 153.0199 | 3.92 | 109.0293, 109.0426, 108.0222, 108.0346, 91.0201 | Negative | 2 | LZ、MZ、P |
| 79 | 1-acetyl-2-hydroxy-4,5-dimethoxybenzene | C_10_H_12_O_4_ | [M+H]^+^ | 7.3284 | 197.0808 | 197.0812 | 2.03 | 140.0469, 155.0703, 123.0442, 125.0232, 151.0753 | Positive | 2 | MZ、P |
| 80 | 1-(2-hydroxy-4,6-dimethoxyphenyl)-2-methoxyethan-1-one | C_11_H_14_O_5_ | [M+H]^+^ | 6.6924 | 227.0914 | 227.0910 | -1.76 | 167.0702, 137.0597, 195.0651, 109.0648, 155.0701 | Positive | 2 | LZ、P |
| 81 | [3,4,5-trihydroxy-6-(hydroxymethyl)oxan-2-yl] 2,4-dihydroxy-6-[(E)-2-phenylethenyl]benzoate | C_21_H_22_O_9_ | [M+H]^+^ | 8.1656 | 419.1337 | 419.1336 | -0.24 | 239.0702, 240.0735, 211.0753, 149.0234, 167.0702 | Positive | 2 | MZ |
| 82 | [(2S,3S,4R,5R)-4-hydroxy-2,5-bis(hydroxymethyl)-2-[(2R,3R,4S,5S,6R)-3,4,5-trihydroxy-6-(hydroxymethyl)oxan-2-yl]oxyoxolan-3-yl] (E)-3-(4-hydroxy-3-methoxyphenyl)prop-2-enoate | C_30_H_30_O_12_ | [M-H]^-^ | 7.652 | 581.1665 | 517.1719 | 9.29 | 175.0401, 193.0507, 160.0164, 89.0245, 341.1068 | Negative | 2 | LZ |
| 83 | (E)-N-[2-hydroxy-2-(4-hydroxyphenyl)ethyl]-3-(4-hydroxy-3-methoxyphenyl)prop-2-enamide | C_18_H_19_NO_5_ | [M+H]^+^ | 6.862 | 330.1336 | 330.1335 | -0.3 | 177.0552, 145.0288, 117.0337, 149.0598, 89.0388 | Positive | 2 | LZ、P |
| 84 | (E)-3-(4-hydroxy-3-methoxyphenyl)prop-2-enamide | C_10_H_11_NO_3_ | [M+H]^+^ | 5.876 | 194.0812 | 194.0812 | 0 | 89.0379, 177.0557, 145.0289, 149.0596, 134.0357 | Positive | 2 | MZ |
| 85 | (E)-3-(4-hydroxy-3-methoxyphenyl)-N-[2-(4-hydroxyphenyl)ethyl]prop-2-enamide | C_18_H_19_NO_4_ | [M+H]^+^ | 8.3975 | 314.1387 | 314.1387 | 0 | 177.0546, 121.0648, 145.0284, 93.0699, 117.0334 | Positive | 2 | LZ、MZ、P |
| 86 | (E)-3-(3,4,5-trimethoxyphenyl)prop-2-enal | C_12_H_14_O_4_ | [M+H]^+^ | 9.5874 | 223.0965 | 223.0965 | 0 | 177.0546, 192.0801, 191.0683, 161.0586, 147.0424 | Positive | 2 | LZ、MZ、P |
| 87 | (2R,3S,4S,5R,6S)-2-(hydroxymethyl)-6-[4-prop-2-enyl-2-[(2S,3R,4S,5S,6R)-3,4,5-trihydroxy-6-(hydroxymethyl)oxan-2-yl]oxyphenoxy]oxane-3,4,5-triol | C_21_H_30_O_12_ | [M+Na]^+^ | 5.8256 | 497.1629 | 497.1620 | -1.81 | 335.1098, 171.0416, 185.0420, 336.1133, 213.3368 | Positive | 2 | LZ、P |
| Coumarins | | | | | | | | | | | |
| 88 | Umbelliferone | C_9_H_6_O_3_ | [M-H]^-^ | 5.8824 | 161.0244 | 161.0252 | 4.97 | 133.0296, 162.0280, 105.0349, 117.0349, 89.0393 | Negative | 2 | LZ、MZ、P |
| 89 | Scopoletin | C_10_H_8_O_4_ | [M+H]^+^ | 6.1719 | 193.0495 | 193.0496 | 0.52 | 133.0285, 178.0253, 122.0363, 150.0312, 94.0425 | Positive | 2 | LZ、MZ、P |
| 90 | Haploperoside C | C_22_H_28_O_13_ | [M+H]^+^ | 5.8387 | 501.1603 | 501.1590 | -2.59 | 193.0494, 355.1018, 85.0286, 71.0494, 129.0545 | Positive | 2 | LZ、MZ |
| 91 | daphnetin | C_9_H_6_O_4_ | [M-H]^-^ | 4.0632 | 177.0193 | 177.0198 | 2.82 | 121.0297, 149.0247, 132.0219, 133.0295, 93.0348 | Negative | 2 | LZ |
| 92 | Citropen | C_11_H_10_O_4_ | [M+H]^+^ | 10.2349 | 207.0652 | 207.0655 | 1.45 | 164.0470, 149.0230, 192.0420, 121.0650, 163.0390 | Positive | 2 | LZ、P |
| 93 | Aesculetin | C_9_H_6_O_4_ | [M-H]^-^ | 5.4363 | 177.0193 | 177.0200 | 3.95 | 133.0294, 149.0245, 105.0350, 121.0295, 89.0402 | Negative | 2 | LZ、MZ、P |
| 94 | 7,8-Dihydroxycoumarin | C_9_H_6_O_4_ | [M-H]^-^ | 5.3295 | 177.0193 | 177.0199 | 3.39 | 121.0287, 105.0323, 133.0285, 149.0257, 132.0204 | Negative | 2 | LZ、MZ、P |
| 95 | 5,6,7-trimethoxychromen-2-one | C_12_H_12_O_5_ | [M+H]^+^ | 8.2721 | 237.0757 | 237.0761 | 1.69 | 207.0282, 222.0511, 193.0485, 178.0250, 191.0331 | Positive | 2 | MZ |
| 96 | 4-Methylesculetin | C_10_H_8_O_4_ | [M-H]^-^ | 6.3832 | 191.0350 | 191.0357 | 3.66 | 119.0502, 147.0452, 103.0553, 93.0345, 95.0502 | Negative | 2 | LZ、MZ、P |
| 97 | 5,6,7-trimethoxychromen-2-one | C_12_H_12_O_5_ | [M+H]^+^ | 8.2721 | 237.0757 | 237.0761 | 1.69 | 207.0282, 222.0511, 193.0485, 178.0250, 191.0331 | Positive | 2 | MZ |
| 98 | 1H-2-benzopyran-1-one, 6,8-dihydroxy-3-methyl- | C_10_H_10_O_4_ | [M-H]^-^ | 8.195 | 193.0506 | 191.0357 | 2.07 | 128.0355, 200.0572, 201.0585, 212.0597, 272.0842 | Negative | 2 | LZ、MZ、P |
| Peptides | | | | | | | | | | |  |
| 99 | Xefoampeptide E | C_26_H_41_N_5_O_6_ | [M+H]^+^ | 16.0577 | 524.3079 | 524.4058 | 186.7 | 496.4103, 252.2315, 245.1858, 298.2374, 280.2262 | Positive | 2 | MZ、P |
| 100 | Xefoampeptide A | C_25_H_39_N_5_O_6_ | [M+H]^+^ | 15.4927 | 510.2922 | 510.3889 | 189.5 | 482.3943, 238.2160, 284.2216, 154.1580, 245.1856 | Positive | 2 | P |
| 101 | Pyrrolo[1,2-a]pyrazine-1,4-dione, hexahydro-3-(1-methylethyl)- | C_10_H_16_N_2_O_2_ | [M+H]^+^ | 5.165 | 197.1285 | 197.1282 | -1.52 | 70.0639, 154.0743, 169.1350, 124.1130, 153.0664 | Positive | 2 | LZ |
| 102 | N-Fructosyl pyroglutamate | C_11_H1_7_NO_8_ | [M-H]^-^ | 0.969 | 290.0881 | 290.0885 | 1.38 | 128.0355, 200.0572, 201.0585, 212.0597, 272.0842 | Negative | 2 | LZ、MZ、P |
| 103 | GameXPeptide C | C_29_H_53_N_5_O_5_ | [M+H]^+^ | 13.4457 | 552.4119 | 552.4102 | -3.08 | 227.1749, 213.1594, 326.2433, 199.1802, 340.2591 | Positive | 2 | P |
| 104 | Cystine | C_6_H_12_N_2_O_4_S_2_ | [M-H]^-^ | 7.7208 | 239.0166 | 239.0219 | 22.1 | 223.0278, 74.9894, 149.0084, 93.0000, 91.0207 | Negative | 2 | MZ |
| 105 | Arenamide A | C_36_H_57_N_5_O_7_ | [M+Na]^+^ | 14.3913 | 694.4150 | 694.4133 | -2.45 | 694.4180, 476.3116, 547.3495, 363.2273, 695.4210 | Positive | 2 | MZ |
| 106 | 3-butan-2-yl-2,3,6,7,8,8a-hexahydropyrrolo[1,2-a]pyrazine-1,4-dione | C_11_H_18_N_2_O_2_ | [M+H]^+^ | 6.114 | 211.1441 | 211.1443 | 0.95 | 70.0654, 154.0756, 138.1308, 183.1519, 86.1004 | Positive | 2 | LZ |
| Nucleosides | | | | | | | | | | | |
| 107 | Adenosine | C_10_H_13_N_5_O_4_ | [M+H]^+^ | 1.7096 | 268.104 | 268.1046 | 2.24 | 136.0618, 119.0352, 137.0456, 94.0399, 71.0127 | Positive | 2 | MZ、P |
| Naphthalenes | | | | | | | | | | | |
| 108 | Tanshinone Iia | C_19_H_18_O_3_ | [M+H]^+^ | 15.5059 | 295.1329 | 295.1323 | -2.03 | 206.1090, 234.1039, 262.0988, 252.0780, 249.1274 | Positive | 2 | MZ |
| 109 | Eleutherol | C_14_H_12_O_4_ | [M+H]^+^ | 10.7094 | 245.0808 | 245.0803 | -2.04 | 128.0630, 184.0522, 156.0574, 155.0500, 139.0549 | Positive | 2 | LZ、P |
| 110 | 1-Naphthol | C_10_H_8_O | [M+H]^+^ | 8.8949 | 145.0648 | 145.0645 | -2.07 | 102.0464, 115.0543, 91.0543, 89.0386, 65.0386 | Positive | 2 | LZ、MZ、P |
| 111 | (1R,2R,4aS,8aS)-2-[(2R)-2-Hydroxybutyl]-1,3-dimethyl-1,2,4a,5,6,7,8,8a-octahydro-1-naphthalenecarboxylic acid | C_17_H_26_O_3_ | [M+H-H2O]^+^ | 13.2981 | 263.2006 | 263.2020 | 5.32 | 119.0851, 161.1326, 217.1949, 133.1010, 147.1165 | Positive | 2 | LZ |
| Macrolides | | | | | | | | | | | |
| 112 | 5,13,15-trihydroxy-9-methyl-10-oxabicyclo[10.4.0]hexadeca-1(12),13,15-triene-3,11-dione | C_16_H_20_O_6_ | [M-H]^-^ | 8.8892 | 307.1187 | 307.1188 | 0.33 | 191.0344, 123.0439, 149.0233, 221.1179, 147.0441 | Negative | 2 | LZ、MZ |
| 113 | [(4E)-7-acetyloxy-6-hydroxy-2-methyl-10-oxo-2,3,6,7,8,9-hexahydrooxecin-3-yl] (E)-but-2-enoate | C_24_H_24_O_10_ | [M-H]^-^ | 8.3027 | 471.1296 | 471.1312 | 3.4 | 145.0284, 163.0391, 146.0317, 119.0490, 265.0719 | Negative | 2 | LZ |
| Lipids | | | | | | | | | | | |
| 114 | Suberic acid | C_8_H_14_O_4_ | [M-H]^-^ | 6.5991 | 173.0819 | 173.0825 | 3.47 | 111.0815, 83.0500, 57.0341, 109.0659, 129.0920 | Negative | 2 | MZ |
| 115 | Stearidonic acid ethyl ester | C_20_H_32_O_2_ | [M+H]^+^ | 14.6152 | 305.2475 | 305.2473 | -0.65 | 93.0702, 135.1168, 107.0858, 121.1013, 81.0704 | Positive | 2 | LZ、P |
| 116 | Sphinganine | C_18_H_39_NO_2_ | [M+H]^+^ | 12.4513 | 302.3054 | 302.3054 | 0 | 60.0443, 284.2944, 254.2839, 95.0851, 81.0698 | Positive | 2 | LZ、MZ、P |
| 117 | Phytosphingosine | C_18_H_39_NO_3_ | [M+H]^+^ | 11.59 | 318.3003 | 318.3003 | 0 | 60.0450, 282.2785, 56.0501, 300.2890, 270.2789 | Positive | 2 | LZ、MZ、P |
| 118 | Phytoceramide C2 | C_20_H_41_NO_4_ | [M+H]^+^ | 14.0142 | 360.3108 | 360.3109 | 0.28 | 60.0449, 342.2998, 282.2788, 264.2684, 86.0602 | Positive | 2 | LZ、P |
| 119 | PE(16:0/18:1) | C_39_H_76_NO_8_P | [M-H]^-^ | 15.973 | 716.5236 | 716.5253 | 2.37 | N/A | Negative | 2 | MZ |
| 120 | PC(19:1/0:0) | C_27_H_54_NO_7_P | [M+H]^+^ | 14.7575 | 536.3711 | 536.3695 | -2.98 | 184.0733, 104.1068, 518.3599, 353.3053, 258.1100 | Positive | 2 | LZ、MZ、P |
| 121 | PC(18:2/0:0) | C_26_H_50_NO_7_P | [M+H]^+^ | 13.2789 | 520.3398 | 520.3384 | -2.69 | 184.0729, 104.1065, 124.9994, 502.3278, 337.2725 | Positive | 2 | LZ、MZ、P |
| 122 | PC(0:0/20:4) | C_28_H_50_NO_7_P | [M+H]^+^ | 14.0723 | 544.3398 | 544.3353 | -8.27 | N/A | Positive | 2 | LZ、MZ、P |
| 123 | PC(0:0/18:1) | C_26_H_52_NO_7_P | [M+H]^+^ | 14.0815 | 522.3554 | 522.3538 | -3.06 | N/A | Positive | 2 | LZ、MZ、P |
| 124 | PC(0:0/18:0) | C_26_H_54_NO_7_P | [M+H]^+^ | 15.0379 | 524.3711 | 524.3697 | -2.67 | N/A | Positive | 2 | LZ、P |
| 125 | PC(0:0/16:0) | C_24_H_50_NO_7_P | [M+H]^+^ | 13.7391 | 496.3398 | 496.3391 | -1.41 | N/A | Positive | 2 | LZ、MZ、P |
| 126 | palmitoleic acid | C_16_H_30_O_2_ | [M+H]^+^ | 13.4281 | 255.2319 | 255.2321 | 0.78 | 69.0703, 57.0704, 83.0859, 219.2109, 55.0548 | Positive | 2 | LZ、MZ、P |
| 127 | Palmitelaidic acid | C_16_H_30_O_2_ | [M+H-H2O]^+^ | 13.4168 | 237.2213 | 237.2214 | 0.42 | 69.0706, 83.0861, 95.0860, 81.0704, 97.1017 | Positive | 2 | LZ、MZ、P |
| 128 | oleic acid | C_18_H_34_O_2_ | [M+H-H2O]^+^ | 17.8321 | 265.2526 | 265.2526 | 0 | 69.0705, 81.0705, 67.0549, 95.0860, 83.0861 | Positive | 2 | LZ、MZ、P |
| 129 | Octanedioic acid | C_8_H_14_O_4_ | [M-H]^-^ | 6.4843 | 173.0819 | 173.0832 | 7.51 | 111.0815, 83.0500, 57.0341, 109.0659, 129.0920 | Negative | 2 | P |
| 130 | N-Oleoylethanolamine | C_20_H_39_NO_2_ | [M+H]^+^ | 15.98 | 326.3054 | 326.3051 | -0.92 | 62.0603, 69.0701, 309.2809, 95.0855, 81.0704 | Positive | 2 | LZ、MZ、P |
| 131 | Monoolein | C^21^H^40^O^4^ | [M+H]^+^ | 16.9888 | 357.2999 | 357.2995 | -1.12 | 265.2526, 247.2420, 339.2893, 121.1015, 95.0861 | Positive | 2 | LZ、P |
| 132 | Monolinolenin (9c,12c,15c) | C^21^H^36^O^4^ | [M+H]^+^ | 12.9456 | 353.2686 | 353.2683 | -0.85 | 81.0703, 95.0859, 67.0548, 121.1012, 109.1013 | Positive | 2 | LZ、MZ、P |
| 133 | LPC 18:2 | C^26^H^50^NO^7^P | [M+H]^+^ | 13.0107 | 520.3398 | 520.3378 | -3.84 | 184.0752, 104.1077, 502.3284, 185.0766, 86.0967 | Positive | 2 | LZ |
| 134 | Linoleoyl ethanolamide | C^20^H^37^NO^2^ | [M+H]^+^ | 14.9632 | 324.2897 | 324.2897 | 0 | 62.0608, 81.0705, 95.0860, 67.0549, 109.1016 | Positive | 2 | LZ、MZ、P |
| 135 | linoleic acid | C^18^H^32^O^2^ | [M+H]^+^ | 13.9892 | 281.2475 | 281.2476 | 0.35 | 91.0541, 105.0697, 81.0697, 67.0541, 93.0697 | Positive | 2 | LZ、MZ、P |
| 136 | Likely Trp-C3:0 + O (hydroxylated) | C^13^H^16^N^2^O^5^ | [M+H]^+^ | 4.9842 | 277.1183 | 277.1184 | 0.36 | 188.0708, 146.0602, 132.0810, 189.0742, 231.1126 | Positive | 2 | LZ |
| 137 | Lauryldiethanolamine | C_16_H_35_NO_2_ | [M+H]^+^ | 10.6435 | 274.2741 | 274.2740 | -0.36 | 70.0651, 88.0757, 57.0699, 106.0863, 102.0914 | Positive | 2 | LZ、MZ、P |
| 138 | L-alpha-Glycerylphosphorylcholine | C_8_H_20_NO_6_P | [M+H]^+^ | 0.884 | 258.1101 | 258.1104 | 1.16 | 104.1067, 124.9996, 86.0962, 184.0733, 60.0806 | Positive | 2 | LZ、MZ、P |
| 139 | Hexopyranoside, 3,4-dimethoxyphenyl 6-O-(6-deoxy-alpha-L-mannopyranosyl)- | C_20_H_30_O_12_ | [M+NH4]^+^ | 5.0097 | 480.2075 | 480.2068 | -1.46 | 155.0712, 85.0281, 129.0554, 147.0647, 71.0490 | Positive | 2 | P |
| 140 | Glycerol 1-stearate | C_21_H_42_O_4_ | [M+H]^+^ | 18.3768 | 359.3156 | 359.3150 | -1.67 | 71.0864, 57.0708, 95.0861, 85.1019, 81.0706 | Positive | 2 | LZ、MZ、P |
| 141 | gamma-linolenic acid | C_18_H_30_O_2_ | [M+H]^+^ | 14.1013 | 279.2319 | 279.2316 | -1.07 | 81.0702, 67.0540, 93.0697, 95.0850, 79.0545 | Positive | 2 | LZ、MZ |
| 142 | GalCer(d18:2/18:1) | C_42_H_77_NO_8_ | [M+H]^+^ | 17.5594 | 724.5722 | 724.5694 | -3.86 | 262.2531, 544.5095, 546.5245, 280.2637, 250.2533 | Positive | 2 | MZ、P |
| 143 | Fisetin 3'4'-Dimethyl Ether | C_17_H_14_O_6_ | [M-H]^-^ | 9.7215 | 313.0712 | 313.0726 | 4.47 | 255.0291, 227.0329, 297.0401, 283.0215, 269.0466 | Negative | 2 | P |
| 144 | FA 18:1+3O | C_18_H_34_O_5_ | [M-H]^-^ | 10.0213 | 329.2334 | 329.2338 | 1.21 | 211.1335, 229.1446, 171.1020, 183.1402, 139.1117 | Negative | 2 | LZ、MZ、P |
| 145 | Citric acid | C_6_H_8_O_7_ | [M-H]^-^ | 0.9727 | 191.0197 | 191.0204 | 3.66 | 87.0106, 111.0099, 85.0312, 67.0223, 57.0391 | Negative | 2 | MZ |
| 146 | cis,cis-9,12-Octadecadien-1-ol | C_18_H_34_O | [M+H]^+^ | 14.5491 | 267.2682 | 267.2684 | 0.75 | 81.0700, 95.0855, 109.1012, 67.0546, 123.1168 | Positive | 2 | LZ、MZ、P |
| 147 | Benzyl 6-O-(6-deoxy-alpha-L-mannopyranosyl)-beta-D-glucopyranoside | C_19_H_28_O_10_ | [M+NH4]^+^ | 5.7032 | 434.2021 | 434.2013 | -1.84 | 85.0273, 129.0540, 71.0481, 91.0541, 147.0618 | Positive | 2 | P |
| 148 | Azelaic acid | C_9_H_16_O_4_ | [M-H]^-^ | 7.4431 | 187.0976 | 187.0984 | 4.28 | 125.0971, 97.0656, 123.0816, 57.0344, 143.1077 | Negative | 2 | LZ、MZ、P |
| 149 | amylamine-C18:0 | C_23_H_47_NO | [M+H]^+^ | 16.3821 | 354.373 | 354.3726 | -1.13 | 284.2946, 88.0757, 102.0913, 57.0703, 71.0858 | Positive | 2 | P |
| 150 | AEG(o-16:2/18:2) | C_37_H_66_O_4_ | [M+H]^+^ | 18.9772 | 575.5034 | 575.4999 | -6.08 | 95.0858, 81.0704, 57.0707, 109.1014, 71.0862 | Positive | 2 | LZ |
| 151 | 9Z,11E,13E-Octadecatrienoic acid methyl ester | C_19_H_32_O_2_ | [M+H]^+^ | 15.4701 | 293.2475 | 293.2473 | -0.68 | 95.0856, 81.0701, 67.0546, 109.1013, 123.1168 | Positive | 2 | LZ |
| 152 | 9-Oxo-10E,12Z-octadecadienoic acid | C_18_H_30_O_3_ | [M+H]^+^ | 14.1639 | 295.2268 | 295.2268 | 0 | 151.1118, 69.0705, 95.0495, 81.0704, 67.0548 | Positive | 2 | LZ、MZ、P |
| 153 | 9,12-Octadecadiynoic Acid | C_18_H_30_O_2_ | [M+H]^+^ | 13.9293 | 277.2162 | 277.2162 | 0 | 93.0702, 107.0857, 81.0703, 79.0547, 121.1012 | Positive | 2 | MZ |
| 154 | 8-{(1S,5R)-4-Oxo-5-[(2Z)-2-penten-1-yl]-2-cyclopenten-1-yl}octanoic acid | C_18_H_28_O_3_ | [M+H-H2O]^+^ | 9.7783 | 275.2006 | 275.2004 | -0.73 | 105.0702, 91.0540, 79.0530, 131.0866, 145.1022 | Positive | 2 | LZ、MZ |
| 155 | 3-Hexen-1-ol O-b-D-glucopyranoside | C_12_H_22_O_6_ | [M+Na]^+^ | 7.9581 | 285.1309 | 285.1315 | 2.1 | 148.0470, 147.0440, 121.0650, 122.0680, 257.1530 | Positive | 2 | LZ、MZ、P |
| 156 | 2,2'-(Tetradecylimino)diethanol | C_18_H_39_NO_2_ | [M+H]^+^ | 11.5444 | 302.3054 | 302.3054 | 0 | 70.0650, 88.0757, 57.0699, 106.0862, 102.0913 | Positive | 2 | LZ、MZ、P |
| 157 | 1-Hexadecanoyl-sn-glycerol | C_19_H_38_O_4_ | [M+H]^+^ | 16.6632 | 331.2843 | 331.284 | -0.91 | 57.0703, 71.0857, 95.0854, 85.1011, 313.2725 | Positive | 2 | LZ、MZ、P |
| 158 | 1-Hexadecanoyl-sn-glycero-3-phosphocholine | C_24_H_50_NO_7_P | [M+Na]^+^ | 13.7559 | 518.3217 | 518.3204 | -2.51 | 104.1070, 459.2506, 146.9816, 86.0964, 313.2735 | Positive | 2 | LZ、MZ、P |
| 159 | 1-Hexadecanoyl-2-(9Z-octadecenoyl)-sn-glycero-3-phosphocholine | C_42_H_82_NO_8_P | [M+Na]^+^ | 17.172 | 782.5670 | 782.5630 | -5.11 | 184.0738, 786.5953, 783.5685, 784.5746, 185.0749 | Positive | 2 | LZ、P |
| 160 | 1-Heptadecanoyl-sn-glycero-3-phosphocholine | C_25_H_52_NO_7_P | [M+H]^+^ | 14.2572 | 510.3554 | 510.3536 | -3.53 | 184.0733, 104.1073, 86.0969, 125.0000, 60.0815 | Positive | 2 | MZ |
| 161 | 15-oxo-11Z,13E-eicosadienoic acid | C_20_H_34_O_3_ | [M+H-H2O]^+^ | 15.1288 | 305.2475 | 305.2467 | -2.62 | 93.0702, 121.1012, 107.0858, 135.1168, 79.0547 | Positive | 2 | LZ、MZ |
| 162 | 13S-Hydroxy-9Z,11E,15Z-octadecatrienoic acid | C_18_H_30_O_3_ | [M+H-H2O]^+^ | 13.5617 | 277.2162 | 277.2162 | 0 | 93.0702, 121.1013, 107.0857, 79.0547, 135.1168 | Positive | 2 | LZ、MZ、P |
| 163 | 13-Keto-9Z,11E-octadecadienoic acid | C_18_H_30_O_3_ | [M+H-H2O]^+^ | 14.034 | 277.2162 | 277.2162 | 0 | 135.1167, 93.0699, 121.1011, 107.0855, 81.0700 | Positive | 2 | LZ、MZ、P |
| 164 | 12-Oxodihydrophytodienoic acid | C_18_H_28_O_3_ | [M+H-H2O]^+^ | 14.3892 | 277.2162 | 277.2161 | -0.36 | 93.0716, 79.0562, 91.0559, 107.0866, 77.0406 | Positive | 2 | LZ、MZ、P |
| 165 | 12(13)-Epoxy-9Z-octadecenoic acid | C_18_H_32_O_3_ | [M+H]^+^ | 13.1853 | 243.2108 | 243.2107 | -0.41 | 173.1329, 242.2488, 187.1486, 159.1172, 145.1015 | Positive | 2 | LZ、MZ、P |
| 166 | 1-(9Z-Octadecenoyl)-sn-glycero-3-phosphocholine | C_26_H_52_NO_7_P | [M+H]^+^ | 14.1952 | 522.3554 | 522.3535 | -3.64 | 184.0731, 104.1072, 86.0968, 60.0814, 124.9998 | Positive | 2 | MZ |
| 167 | linolenic acid | C_18_H_30_O_2_ | [M+H]^+^ | 13.7682 | 279.2319 | 279.2319 | 0 | 67.0536, 81.0692, 95.0852, 79.0541, 65.0381 | Positive | 2 | LZ、MZ、P |
| Heterocyclic Compounds | | | | | | | | | | | |
| 168 | 5-phenyl-1,3-oxazolidin-2-one | C_9_H_9_NO_2_ | [M+NH4]^+^ | 6.6759 | 181.0977 | 181.1010 | 18.22 | 165.0696, 115.0539, 164.0608, 178.0791, 128.0617 | Positive | 2 | MZ、P |
| Lignans | | | | | | | | | | | |
| 169 | syringaresinol | C_22_H_26_O_8_ | [M-H]^-^ | 8.8698 | 417.1555 | 417.1559 | 0.96 | 166.0268, 181.0518, 387.1060, 137.0208, 167.0353 | Negative | 2 | LZ、MZ、P |
| 170 | Secoisolariciresinol | C_20_H_26_O_6_ | [M-H]^-^ | 7.5956 | 361.1656 | 361.1657 | 0.28 | 361.1637, 362.1667, 165.0518, 346.1465, 122.0360 | Negative | 2 | LZ、MZ、P |
| 171 | Pinoresinol | C_20_H_22_O_6_ | [M-H]^-^ | 9.1104 | 357.1344 | 357.1343 | -0.28 | 136.0164, 151.0400, 121.0295, 122.0375, 137.0218 | Negative | 2 | LZ、MZ、P |
| 172 | isolariciresinol | C_20_H_26_O_6_ | [M-H]^-^ | 6.9374 | 359.1500 | 359.1501 | 0.28 | 344.1261, 313.1075, 299.0918, 241.0498, 343.1184 | Negative | 2 | LZ、MZ、P |
| 173 | Grossamide | C_36_H_34_N_2_O_8_ | [M+H]^+^ | 10.309 | 623.2388 | 625.2529 | 3.22 | 325.1073, 351.0883, 307.0966, 462.1897, 326.1071 | Positive | 2 | MZ |
| 174 | Episyringaresinol 4'-O-beta-D-glncopyranoside | C_28_H_36_O_13_ | [M+Na]^+^ | 7.0271 | 603.2048 | 603.2036 | -1.99 | 440.1440, 185.0420, 441.1520, 425.1200, 309.0940 | Positive | 2 | LZ、MZ、P |
| 175 | Eleutheroside E | C_34_H_46_O_18_ | [M+Na]^+^ | 5.9986 | 765.2576 | 765.2567 | -1.18 | 603.2050, 441.1520, 440.1440, 185.0420, 602.1970 | Positive | 2 | LZ、MZ、P |
| 176 | 6,7-bis(hydroxymethyl)-1-methoxy-8-(3,4,5-trimethoxyphenyl)-5,6,7,8-tetrahydronaphthalene-2,3-diol | C_21_H_26_O_8_ | [M+FA-H]^-^ | 6.5867 | 465.1766 | 465.1761 | -1.08 | 419.1707, 404.1424, 373.1371, 389.1209, 358.1086 | Negative | 2 | LZ、MZ、P |
| 177 | 5-[6-(3-hydroxy-4-methoxyphenyl)-1,3,3a,4,6,6a-hexahydrofuro[3,4-c]furan-3-yl]-2-methoxyphenol | C_20_H_22_O_6_ | [M+H-H2O]^+^ | 9.13 | 341.1384 | 341.1382 | -0.59 | 137.0592, 187.0739, 291.1028, 211.0757, 270.0875 | Positive | 2 | LZ、MZ、P |
| 178 | 4-[(3R,3aR,6S,6aR)-6-(4-hydroxy-3,5-dimethoxyphenyl)-1,3,3a,4,6,6a-hexahydrofuro[3,4-c]furan-3-yl]-2,6-dimethoxyphenol | C_22_H_26_O_8_ | [M+H]^+^ | 8.8843 | 419.1700 | 419.1691 | -2.15 | 145.0662, 173.0612, 217.0867, 167.0734, 205.0877 | Positive | 2 | LZ、MZ、P |
| 179 | 3,4,5-Trimethoxybenzaldehyde | C_10_H_12_O_4_ | [M+H]^+^ | 8.9193 | 197.0808 | 197.0797 | -5.58 | 169.0859, 138.0676, 154.0624, 123.0440, 139.0390 | Positive | 2 | MZ |
| 180 | 2-[[5-(4-hydroxy-3,5-dimethoxyphenyl)-6,7-bis(hydroxymethyl)-1,3-dimethoxy-5,6,7,8-tetrahydronaphthalen-2-yl]oxy]-6-(hydroxymethyl)oxane-3,4,5-triol | C_28_H_38_O_13_ | [M+NH4]^+^ | 5.4768 | 600.2651 | 600.2641 | -1.67 | 249.1130, 250.1159, 187.0760, 267.1239, 159.0814 | Positive | 2 | P |
| 181 | 2,3-bis[(4-hydroxy-3-methoxyphenyl)methyl]butane-1,4-diol | C_20_H_26_O_6_ | [M+H-2H2O]^+^ | 7.6212 | 327.1591 | 327.159 | -0.31 | 137.0597, 163.0753, 133.0649, 131.0492, 295.1329 | Positive | 2 | LZ、MZ、P |
| 182 | Liriodendrin | C_34_H_46_O_18_ | [M+NH4]^+^ | 6.0179 | 760.3022 | 760.3017 | -0.66 | 205.0860, 265.1078, 167.0702, 173.0596, 217.0863 | Positive | 2 | L、P |
| 183 | (2S,3R,4S,5S,6R)-2-[4-[(3S,3aR,6S,6aR)-3-(4-hydroxy-3,5-dimethoxyphenyl)-1,3,3a,4,6,6a-hexahydrofuro[3,4-c]furan-6-yl]-2,6-dimethoxyphenoxy]-6-(hydroxymethyl)oxane-3,4,5-triol | C_28_H_36_O_13_ | [M+NH4]^+^ | 7.0101 | 598.2494 | 598.2485 | -1.51 | 205.0856, 167.0703, 173.0604, 265.1072, 217.0854 | Positive | 2 | LZ、P |
| 184 | (1R,2S)-7-hydroxy-1-(4-hydroxy-3,5-dimethoxyphenyl)-2-N,3-N-bis[2-(4-hydroxyphenyl)ethyl]-6,8-dimethoxy-1,2-dihydronaphthalene-2,3-dicarboxamide | C_38_H_40_N_2_O_10_ | [M+H]^+^ | 9.0854 | 685.2756 | 685.2745 | -1.6 | 351.0872, 548.1942, 383.1139, 231.0656, 520.1987 | Positive | 2 | LZ、MZ、P |
| Flavonoids and their derivatives | | | | | | | | | | | |
| 185 | Tectorigenin | C_16_H_12_O_6_ | [M-H]^-^ | 6.3032 | 299.0561 | 299.0564 | 1 | 284.0314, 240.0428, 255.0290, 283.0211, 227.0336 | Negative | 2 | LZ、MZ、P |
| 186 | 2,3-Dimethoxyxanthen-9-one | C_15_H_12_O_4_ | [M+H]^+^ | 8.8189 | 257.0808 | 257.0812 | 1.56 | 213.0559, 241.0507, 214.0584, 168.0569, 242.0566 | Positive | 2 | LZ、P |
| 187 | Mangiferin | C_19_H_18_O_11_ | [M-H]^-^ | 5.5063 | 421.0776 | 421.0780 | 0.95 | 301.0343, 331.0446, 375.0659, 259.0228, 271.0226 | Negative | 2 | MZ、P |
| 188 | Genistein | C_15_H_10_O_5_ | [M-H]^-^ | 6.5138 | 269.0455 | 269.0462 | 2.6 | 225.0559, 181.0659, 201.0557, 197.0607, 224.0476 | Negative | 2 | LZ、MZ、P |
| 189 | Equol | C_15_H_14_O_3_ | [M+H]^+^ | 10.4614 | 243.1016 | 243.1009 | -2.88 | 133.0630, 107.0484, 123.0434, 105.0692, 134.0683 | Positive | 2 | LZ、P |
| 190 | Daidzein | C_15_H_10_O_4_ | [M+H]^+^ | 9.4774 | 255.0652 | 255.0653 | 0.39 | 181.0634, 199.0740, 137.0223, 152.0604, 153.0682 | Positive | 2 | LZ、MZ、P |
| 191 | biochanin A | C_16_H_12_O_5_ | [M-H]^-^ | 8.18 | 283.0612 | 283.0618 | 2.12 | 211.0400, 239.0348, 267.0296, 132.0214, 195.0450 | Negative | 2 | LZ、MZ、P |
| 192 | Biochanin | C_16_H_12_O_5_ | [M+H]^+^ | 7.5315 | 285.0757 | 285.0759 | 0.7 | 242.0600, 152.0090, 213.0570, 269.0500, 253.0540 | Positive | 2 | LZ、MZ、P |
| 193 | 1,6-dihydroxy-3-methoxy-8-methylxanthen-9-one | C_15_H_12_O_5_ | [M+H]^+^ | 12.1854 | 273.0757 | 273.0759 | 0.73 | 230.0581, 212.0471, 258.0524, 229.0498, 274.0800 | Positive | 2 | LZ、P |
| 194 | (S)-3-(4-hydroxyphenyl)chroman-7-ol | C_15_H_14_O_3_ | [M+H]^+^ | 9.9658 | 243.1016 | 243.1016 | 0 | 105.0700, 107.0490, 242.0840, 133.0650, 103.0540 | Positive | 2 | LZ、MZ、P |
| 195 | Techtochrysin | C_16_H_12_O_4_ | [M+H]^+^ | 6.7169 | 269.0808 | 269.0810 | 0.74 | 226.0542, 124.0116, 197.0544, 152.0561, 225.0485 | Positive | 2 | LZ、MZ、P |
| 196 | taxifolin | C_15_H_12_O_7_ | [M+H]^+^ | 6.1848 | 305.0656 | 305.0654 | -0.66 | 153.0180, 231.0651, 149.0235, 123.0440, 259.0594 | Positive | 2 | M、P |
| 197 | Tangeretin | C_20_H_20_O_7_ | [M+H]^+^ | 11.9235 | 373.1282 | 373.1274 | -2.14 | 343.0806, 297.0753, 183.0286, 300.0623, 211.0234 | Positive | 2 | L、MZ |
| 198 | 2-(3,4-Dihydroxybenzylidene)-6-hydroxybenzofuran-3(2H)-one | C_15_H_10_O_5_ | [M-H]^-^ | 7.9104 | 269.0455 | 269.0462 | 2.6 | N/A | Negative | 2 | LZ、MZ、P |
| 199 | SNG | C_28_H_26_O_14_ | [M-H]^-^ | 6.7368 | 577.1555 | 577.1569 | 2.43 | N/A | Negative | 2 | MZ |
| 200 | SAKURANETIN | C_16_H_14_O_5_ | [M-H]^-^ | 10.4024 | 285.0768 | 285.0776 | 2.81 | 165.0183, 119.0489, 93.0331, 191.0341, 145.0286 | Negative | 2 | MZ、P |
| 201 | Kaempferol-3-O-rhamnoside-7-O-rhamnoside | C_27_H_30_O_14_ | [M+H]^+^ | 6.9095 | 579.1708 | 579.1690 | -3.11 | 287.0545, 433.1110, 85.0294, 71.0508, 129.0546 | Positive | 2 | MZ |
| 202 | Quercitrin | C_21_H_20_O_11_ | [M+H]^+^ | 7.1172 | 449.1078 | 447.0929 | -3.32 | 303.0499, 287.0549, 304.0523, 85.0281, 153.0181 | Positive | 2 | M、P |
| 203 | Quercetin-3-O-glucoside | C_21_H_20_O_12_ | [M-H]^-^ | 6.6448 | 463.0882 | 463.0882 | 0 | N/A | Negative | 2 | MZ |
| 204 | Quercetin-3,7-O-alpha-L-dirhamnopyranoside | C_27_H_30_O_15_ | [M+H]^+^ | 6.3762 | 595.1658 | 593.1509 | -2.51 | N/A | Positive | 2 | MZ |
| 205 | Quercetin | C_15_H_10_O_7_ | [M+H]^+^ | 7.4945 | 303.0499 | 303.0495 | -1.32 | 153.0180, 229.0496, 137.0230, 257.0447, 201.0546 | Positive | 2 | MZ |
| 206 | Naringenin-7-O-glucoside | C_21_H_22_O_10_ | [M-H]^-^ | 7.1532 | 433.1140 | 433.1137 | -0.69 | 271.0616, 151.0038, 119.0503, 177.0195, 93.0345 | Negative | 2 | P |
| 207 | Pinocembrine | C_15_H_12_O_4_ | [M+H]^+^ | 11.0282 | 257.0808 | 257.0809 | 0.39 | 153.0182, 131.0491, 103.0542, 79.0545, 107.0490 | Positive | 2 | LZ、MZ、P |
| 208 | pinocembrin | C_15_H_12_O_4_ | [M-H]^-^ | 11.0244 | 255.0663 | 255.0670 | 2.74 | 153.0182, 131.0491, 103.0542, 79.0545, 107.0490 | Negative | 2 | LZ、MZ、P |
| 209 | naringenin | C_15_H_12_O_5_ | [M+H]^+^ | 7.4368 | 273.0758 | 273.0758 | 0 | 137.0233, 163.0389, 135.0439, 145.0282, 117.0334 | Positive | 2 | MZ、P |
| 210 | licochalcone B | C_16_H_14_O_5_ | [M-H]^-^ | 8.3891 | 285.0768 | 285.0774 | 2.11 | 150.0312, 149.0233, 121.0283, 151.0345, 177.0185 | Negative | 2 | LZ、MZ、P |
| 211 | Galangin 3-methyl ether | C_16_H_12_O_5_ | [M+H]^+^ | 8.7741 | 285.0758 | 285.0759 | 0.35 | N/A | Positive | 2 | MZ |
| 212 | Kaempferol-7-O-rhamnoside | C_21_H_20_O_10_ | [M+H]^+^ | 6.9095 | 433.1130 | 433.1130 | 0 | 287.0552, 288.0596, 289.0566, 153.0138, 165.0178 | Positive | 2 | MZ |
| 213 | Isoliquiritigenin | C_15_H_12_O_4_ | [M+H]^+^ | 8.4325 | 257.0808 | 257.0809 | 0.39 | 137.0241, 147.0452, 119.0497, 91.0549, 81.0331 | Positive | 2 | LZ、MZ、P |
| 214 | Homobutein | C_16_H_14_O_5_ | [M+H]^+^ | 8.6817 | 287.0914 | 287.0915 | 0.35 | 137.0234, 145.0284, 177.0545, 117.0334, 163.0389 | Positive | 2 | LZ、MZ、P |
| 215 | Genkwanin | C_16_H_12_O_5_ | [M+H]^+^ | 8.2323 | 285.0758 | 285.0757 | -0.35 | 242.0569, 270.0517, 167.0337, 119.0490, 124.0153 | Positive | 2 | LZ、MZ、P |
| 216 | Eupatorin | C_18_H_16_O_7_ | [M+H]^+^ | 10.994 | 345.0969 | 345.0971 | 0.58 | 284.0680, 312.0630, 269.0440, 330.0730, 148.0520 | Positive | 2 | MZ |
| 217 | Chrysoeriol | C_16_H_12_O_6_ | [M-H]^-^ | 9.6904 | 299.0561 | 299.0566 | 1.67 | 284.0327, 256.0378, 151.0038, 227.0350, 255.0299 | Negative | 2 | LZ、P |
| 218 | Chrysin | C_15_H_10_O_4_ | [M+H]^+^ | 8.8892 | 255.0652 | 255.0652 | 0 | 153.0180, 103.0540, 129.0330, 147.0440, 105.0330 | Positive | 2 | LZ、MZ、P |
| 219 | butein | C_15_H_12_O_5_ | [M+H]^+^ | 9.3474 | 273.0758 | 273.0760 | 0.73 | 137.0233, 163.0389, 135.0440, 145.0284, 117.0334 | Positive | 2 | LZ、MZ、P |
| 220 | Avicularin | C_20_H_18_O_11_ | [M-H]^-^ | 6.9715 | 433.0776 | 433.0784 | 1.85 | 270.0491, 151.0040, 117.0350, 149.0252, 225.0557 | Negative | 2 | MZ、P |
| 221 | Apigeninidin | C_15_H_11_O_4_ | [M]^+^ | 6.9454 | 255.0652 | 255.0651 | -0.39 | 171.0432, 157.0629, 227.0703, 152.0626, 181.0660 | Positive | 2 | MZ、P |
| 222 | Apigenin | C_15_H_10_O_5_ | [M+H]^+^ | 9.5332 | 271.0601 | 271.0605 | 1.47 | 171.0432, 157.0629, 227.0703, 152.0626, 181.0660 | Positive | 2 | LZ、MZ、P |
| 223 | Alpinetin | C_16_H_14_O_4_ | [M+H]^+^ | 9.7561 | 271.0965 | 271.0965 | 0 | 167.0341, 103.0543, 152.0105, 131.0492, 124.0155 | Positive | 2 | LZ、MZ、P |
| 224 | 8-desmethyl-sideroxylin | C_17_H_14_O_5_ | [M+H]^+^ | 8.9981 | 299.0914 | 299.0911 | -1 | 284.0670, 283.0594, 300.0941, 256.0724, 285.0707 | Positive | 2 | LZ、MZ |
| 225 | 7-O-Methylchrysin | C_16_H_12_O_4_ | [M+H]^+^ | 9.485 | 269.0808 | 269.0808 | 0 | 226.0630, 124.0160, 197.0600, 254.0580, 167.0340 | Positive | 2 | LZ、MZ、P |
| 226 | 7-methoxy-2-phenyl-4H-chromen-4-one | C_16_H_12_O_3_ | [M+H]^+^ | 11.3863 | 253.0860 | 253.0860 | 0 | 210.0680, 108.0210, 129.0340, 238.0630, 151.0390 | Positive | 2 | LZ、MZ、P |
| 227 | 7-hydroxyflavone | C_15_H_10_O_3_ | [M+H]^+^ | 9.9069 | 239.0703 | 239.0704 | 0.42 | 129.0338, 137.0237, 68.9981, 103.0549, 65.0395 | Positive | 2 | LZ、MZ、P |
| 228 | 7-Hydroxyflavanone | C_15_H_12_O_3_ | [M+H]^+^ | 10.4173 | 241.0859 | 241.0860 | 0.41 | 137.0233, 131.0491, 103.0542, 195.0803, 163.0388 | Positive | 2 | LZ、MZ、P |
| 229 | 7-hydroxy-2-(4-hydroxyphenyl)-5-methoxy-2,3-dihydrochromen-4-one | C_16_H_14_O_5_ | [M+H]^+^ | 7.3954 | 287.0914 | 287.0910 | -1.39 | 167.0344, 152.0109, 124.0159, 119.0495, 91.0551 | Positive | 2 | LZ |
| 230 | 7-hydroxy-2-(4-hydroxyphenyl)-5-methoxy-2,3-dihydro-4h-chromen-4-one | C_16_H_14_O_5_ | [M+H]^+^ | 7.506 | 287.0914 | 287.0914 | 0 | 167.0344, 152.0109, 124.0159, 119.0495, 91.0551 | Positive | 2 | LZ、MZ、P |
| 231 | 7,8,2'-Trihydroxyflavone | C_15_H_10_O_5_ | [M+H]^+^ | 6.9587 | 271.0601 | 271.0606 | 1.84 | 145.0285, 121.0285, 153.0183, 107.0128, 243.0650 | Positive | 2 | LZ、MZ、P |
| 232 | 7,4'-Dihydroxyflavone | C_15_H_10_O_4_ | [M-H]^-^ | 7.9096 | 253.0506 | 253.0516 | 3.95 | 117.0334, 91.0177, 135.0076, 133.0283, 118.0367 | Negative | 2 | LZ、MZ、P |
| 233 | 7,3'-Dimethoxyflavone | C_17_H_14_O_4_ | [M+H]^+^ | 11.3943 | 283.0965 | 283.0969 | 1.41 | 240.0779, 268.0727, 151.0389, 159.0440, 108.0206 | Positive | 2 | P |
| 234 | 7,3',4'-Trihydroxyflavone | C_15_H_10_O_5_ | [M-H]^-^ | 8.1437 | 269.0455 | 269.0461 | 2.23 | 133.0278, 135.0069, 241.0487, 91.0176, 225.0536 | Negative | 2 | LZ、MZ、P |
| 235 | 6-desmethyl-sideroxylin | C_16_H_12_O_5_ | [M+H]^+^ | 7.2492 | 299.0914 | 299.0912 | -0.67 | 284.0671, 256.0722, 255.0646, 300.0940, 283.0594 | Positive | 2 | LZ、MZ、P |
| 236 | 5-Hydroxy-7-methoxyflavanone | C_16_H_14_O_4_ | [M+H]^+^ | 13.0706 | 271.0965 | 271.0966 | 0.37 | 167.0347, 131.0501, 103.0550, 168.0402, 111.0443 | Positive | 2 | LZ |
| 237 | 5-hydroxy-2-(4-hydroxyphenyl)-7-methoxy-4H-chromen-4-one | C_16_H_12_O_5_ | [M+H]^+^ | 7.2616 | 285.0758 | 285.0757 | -0.35 | 242.0570, 124.0150, 213.0540, 197.0600, 270.0520 | Positive | 2 | LZ |
| 238 | 5,4'-Dihydroxy-7-methoxyflavanone | C_16_H_14_O_5_ | [M+H]^+^ | 9.9532 | 287.0914 | 287.0915 | 0.35 | 167.0328, 147.0430, 119.0482, 111.0427, 168.0357 | Positive | 2 | LZ、MZ、P |
| 239 | 5-hydroxy-2-(4-hydroxyphenyl)-3-[(2S,3R,4S,5S,6R)-3,4,5-trihydroxy-6-(hydroxymethyl)oxan-2-yl]oxy-7-[(2S,3R,4R,5R,6S)-3,4,5-trihydroxy-6-methyloxan-2-yl]oxychromen-4-one | C_27_H_30_O_16_ | [M-H]^-^ | 6.2086 | 609.1461 | 593.1512 |  | N/A | Negative | 2 | MZ |
| 240 | 5,7-Dimethoxy-4'-hydroxyflavanone | C_17_H_16_O_5_ | [M+H]^+^ | 9.6874 | 301.1071 | 301.1071 | 0 | 181.0494, 147.0439, 182.0529, 119.0493, 64.0027 | Positive | 2 | LZ、MZ、P |
| 241 | 5,7-dimethoxy-2-phenyl-4H-chromen-4-one | C_17_H_14_O_4_ | [M+H]^+^ | 7.624 | 283.0965 | 283.0961 | -1.41 | 268.0730, 239.0700, 267.0650, 238.0620, 225.0550 | Positive | 2 | P |
| 242 | 5,6,7,8-tetramethoxy-2-(4-methoxyphenyl)chromen-4-one [IIN-based: Match] | C_20_H_20_O_7_ | [M+H]^+^ | 11.624 | 373.1282 | 373.1273 | -2.41 | 343.0814, 358.1049, 325.0709, 297.0760, 328.0579 | Positive | 2 | MZ |
| 243 | 5,4'-dihydroxy-6-C-methyl-7-methoxy-flavanone | C_17_H_16_O_5_ | [M-H]^-^ | 8.6527 | 299.0925 | 299.0929 | 1.34 | 119.0502, 179.0350, 164.0115, 93.0346, 79.0189 | Negative | 2 | LZ、MZ、P |
| 244 | 4'-Hydroxy-7-methoxyflavone | C_16_H_12_O_4_ | [M+H]^+^ | 9.9405 | 269.0808 | 269.0809 | 0.37 | 226.0620, 254.0568, 151.0387, 145.0282, 108.0204 | Positive | 2 | LZ、MZ、P |
| 245 | 4-Hydroxy-2',4',6'-trimethoxychalcone | C_18_H_18_O_5_ | [M+H]^+^ | 10.3076 | 315.1227 | 315.1224 | -0.95 | 195.0651, 180.0416, 147.0440, 152.0467, 119.0491 | Positive | 2 | P |
| 246 | 4',7-dihydroxyflavone | C_15_H_10_O_4_ | [M+H]^+^ | 7.938 | 255.0652 | 255.0654 | 0.78 | 137.0230, 145.0284, 119.0490, 91.0540, 155.0340 | Positive | 2 | LZ、MZ、P |
| 247 | 4,5-Dihydroxy-7-methoxyflavanone | C_16_H_14_O_5_ | [M+H]^+^ | 10.3272 | 287.0914 | 287.0917 | 1.04 | 167.0337, 147.0437, 119.0490, 168.0379, 148.0490 | Positive | 2 | LZ、MZ、P |
| 248 | 3-deoxysappanchalcone | C_16_H_14_O_4_ | [M-H]^-^ | 9.4618 | 269.0819 | 269.0826 | 2.6 | 120.0203, 108.0203, 237.0551, 161.0233, 92.0254 | Negative | 2 | LZ、MZ、P |
| 249 | 3-{2,4-dihydroxy-5-[3-(4-hydroxy-2-methoxyphenyl)-1-(4-hydroxyphenyl)propyl]phenyl}-1-(4-hydroxyphenyl)propan-1-one | C_31_H_30_O_7_ | [M+H-H2O]^+^ | 9.9199 | 497.1959 | 497.1950 | -1.81 | 137.0594, 107.0492, 241.0850, 119.0492, 239.0696 | Positive | 2 | LZ、MZ、P |
| 250 | 3,3'-Dihydroxyflavone | C_15_H_10_O_4_ | [M-H]^-^ | 9.4576 | 253.0506 | 253.0511 | 1.98 | N/A | Negative | 2 | LZ、MZ、P |
| 251 | 3,3',4'-Trimethoxyflavone | C_18_H_16_O_5_ | [M+H]^+^ | 13.0218 | 313.1071 | 313.1069 | -0.64 | 298.0828, 283.0594, 297.0751, 255.0647, 267.0641 | Positive | 2 | MZ |
| 252 | 2-[[(2R,3S)-2-(3,4-dihydroxyphenyl)-3,5-dihydroxy-3,4-dihydro-2H-chromen-7-yl]oxy]oxane-3,4,5-triol | C_20_H_22_O_10_ | [M-H]^-^ | 5.6715 | 421.1140 | 421.1140 | 0 | 289.0711, 245.0812, 137.0237, 125.0225, 203.0705 | Negative | 2 | MZ、P |
| 253 | 2',6'-dihydroxy-4'-methoxydihydrochalcone | C_16_H_16_O_4_ | [M+H]^+^ | 12.1695 | 273.1121 | 273.1121 | 0 | 91.0481, 105.0631, 141.0469, 115.0469, 255.0936 | Positive | 2 | LZ、MZ |
| 254 | 2,4-Dimethoxy-2'-hydroxychalcone | C_17_H_16_O_4_ | [M+H]^+^ | 10.9987 | 285.1121 | 285.1121 | 0 | 121.0294, 243.1033, 191.0706, 107.0483, 176.0459 | Positive | 2 | LZ、MZ、P |
| 255 | 2',4'-Dihydroxychalcone | C_15_H_12_O_3_ | [M-H]^-^ | 11.6749 | 239.0714 | 239.0726 | 5.02 | N/A | Negative | 2 | LZ、P |
| 256 | 2',3'-Dimethoxyflavanone | C_17_H_16_O_4_ | [M+H]^+^ | 11.1375 | 285.1121 | 285.1124 | 1.05 | 121.0283, 147.0439, 107.0490, 191.0700, 137.0596 | Positive | 2 | MZ |
| 257 | 2-(3,4-dihydroxyphenyl)-7-hydroxy-2,3-dihydrochromen-4-one | C_15_H_12_O_5_ | [M-H]^-^ | 9.3197 | 271.0612 | 271.0620 | 2.95 | N/A | Negative | 2 | LZ、MZ、P |
| 258 | 2-(3,4-dihydroxyphenyl)-5,7-dihydroxy-3-[(2S,3R,4R,5R,6S)-3,4,5-trihydroxy-6-methyloxan-2-yl]oxychromen-4-one | C_21_H_20_O_11_ | [M+H]^+^ | 7.5315 | 449.1078 | 449.1092 | 3.12 | 303.0499, 287.0549, 304.0523, 85.0281, 153.0181 | Positive | 2 | MZ |
| 259 | (E)-3-(3,4-dihydroxy-2-methoxyphenyl)-1-(4-hydroxyphenyl)prop-2-en-1-one | C_16_H_14_O_5_ | [M+H]^+^ | 8.4303 | 287.0914 | 287.0913 | -0.35 | 121.0293, 245.0819, 193.0499, 150.0315, 178.0262 | Positive | 2 | LZ、MZ、P |
| 260 | (E)-1-(4-hydroxy-2-methoxyphenyl)-3-(4-hydroxyphenyl)prop-2-en-1-one | C_16_H_14_O_4_ | [M+H]^+^ | 9.4845 | 271.0965 | 271.0966 | 0.37 | 121.0293, 229.0856, 134.0354, 177.0544, 107.0487 | Positive | 2 | LZ、MZ、P |
| 261 | (2R,3R)-3,5-dihydroxy-2-(4-hydroxyphenyl)-7-methoxy-2,3-dihydrochromen-4-one | C_16_H_14_O_6_ | [M+H]^+^ | 8.6718 | 303.0863 | 303.0864 | 0.33 | 229.0867, 167.0347, 107.0495, 257.0821, 163.0394 | Positive | 2 | LZ、MZ、P |
| 262 | (2E)-1-(2H,3H-benzo[e]1,4-dioxin-6-yl)-3-(7-methoxy(2H-benzo[3,4-d]1,3-dioxole n-5-yl))prop-2-en-1-one | C_19_H_16_O_6_ | [M+H]^+^ | 10.9716 | 341.1020 | 341.1015 | -1.47 | 205.0496, 175.0390, 189.0546, 163.0390, 147.0440 | Positive | 2 | LZ、MZ、P |
| 263 | (2E)-1-(2,4-dihydroxyphenyl)-3-(4-hydroxyphenyl)-2-propen-1-one | C_15_H_12_O_4_ | [M+H]^+^ | 10.0837 | 257.0808 | 257.0817 | 3.5 | 137.0230, 147.0440, 119.0490, 91.0540, 81.0330 | Positive | 2 | LZ、MZ、P |
| 264 | Loureirin A | C_17_H_18_O_4_ | [M+Na]^+^ | 11.408 | 309.1097 | 309.1094 | -0.97 | N/A | Positive | 2 | LZ、MZ、P |
| 265 | Loureirin B | C_18_H_20_O_5_ | [M+Na]^+^ | 13.685 | 339.1203 | 339.1198 | -1.47 | N/A | Positive | 2 | LZ、MZ、P |
| Cabohydrates and glycosides | | | | | | | | | | | |
| 266 | Uridine 5'-diphospho-N-acetylglucosamine | C_17_H_27_N_3_O_17_P_2_ | [M-H]^-^ | 1.2269 | 606.0743 | 606.0751 | 1.32 | 78.9605, 158.9266, 384.9873, 282.0413, 272.9599 | Negative | 2 | MZ |
| 267 | sucrose | C_12_H_22_O_11_ | [M+Na]^+^ | 0.9011 | 365.1054 | 365.1054 | 0 | 203.0521, 185.0417, 204.0557, 366.1078, 202.0593 | Positive | 2 | LZ、MZ、P |
| 268 | Stachyose | C_24_H_42_O_21_ | [M+Na]^+^ | 0.8949 | 689.2059 | 689.2088 | 4.21 | 527.1577, 347.0958, 365.1065, 509.1495, 185.0407 | Positive | 2 | P |
| 269 | Sorbitol | C_6_H_14_O_6_ | [M-H]^-^ | 0.929 | 181.0718 | 181.0724 | 3.31 | 59.0136, 71.0136, 89.0242, 101.0242, 73.0293 | Negative | 2 | LZ、MZ、P |
| 270 | Raffinose | C_18_H_32_O_16_ | [M+Cl]^-^ | 0.9295 | 539.1385 | 539.1389 | 0.74 | N/A | Negative | 2 | LZ、MZ、P |
| 271 | Palatinose | C_12_H_22_O_11_ | [M+Cl]^-^ | 0.9307 | 377.0856 | 377.0859 | 0.8 | N/A | Negative | 2 | LZ、MZ、P |
| 272 | Melibiose | C_12_H_22_O_11_ | [M+HCOO]^-^ | 0.9553 | 387.1144 | 387.1144 | 0 | N/A | Negative | 2 | LZ、MZ、P |
| 273 | maltotriose | C_18_H_32_O_16_ | [M+Na]^+^ | 0.924 | 527.1583 | 527.1573 | -1.9 | 365.1051, 347.0945, 203.0526, 467.1370, 366.1085 | Positive | 2 | LZ、P |
| 274 | Maltitol | C_12_H_24_O_11_ | [M-H]^-^ | 0.8868 | 343.1246 | 343.1246 | 0 | 59.0136, 89.0242, 71.0136, 179.0560, 119.0348 | Negative | 2 | LZ、P |
| 275 | Lactulose | C_12_H_22_O_11_ | [M+H-H2O]^+^ | 0.8285 | 325.1133 | 325.1133 | 0 | 85.0285, 127.0389, 145.0495, 97.0284, 91.0390 | Positive | 2 | MZ |
| 276 | D-Mannitol | C_6_H_14_O_6_ | [M-H+FA]^-^ | 0.8369 | 227.0772 | 227.078 | 3.52 | 181.0712, 101.0232, 89.0232, 71.0126, 59.0126 | Negative | 2 | P |
| 277 | D-Arabitol | C_5_H_12_O_5_ | [M-H]^-^ | 0.9265 | 151.0612 | 151.0616 | 2.65 | 71.0125, 59.0124, 101.0231, 55.0175, 73.0281 | Negative | 2 | LZ、MZ、P |
| 278 | alpha,alpha-Trehalose | C_12_H_22_O_11_ | [M+NH4]^+^ | 0.9245 | 360.1500 | 360.1501 | 0.28 | 85.0281, 145.0494, 163.0600, 127.0387, 97.0281 | Positive | 2 | LZ、MZ、P |
| 279 | Adonitol | C_5_H_12_O_5_ | [M-H]^-^ | 0.8578 | 151.0612 | 151.0619 | 4.63 | 71.0125, 59.0125, 101.0232, 55.0176, 83.0125 | Negative | 2 | MZ、P |
| 280 | (2R,3S,4S,5R,6S)-2-[[(2S,3R,4R)-3,4-dihydroxy-4-(hydroxymethyl)oxolan-2-yl]oxymethyl]-6-(3,4,5-trimethoxyphenoxy)oxane-3,4,5-triol | C_20_H_30_O_13_ | [M+Na]^+^ | 5.3088 | 501.1579 | 501.1569 | -2 | 185.0810, 153.0549, 154.0623, 209.0823, 125.0597 | Positive | 2 | LZ、MZ、P |
| 281 | (2R,3S,4S,5R,6S)-2-[(3,4,5-trihydroxyoxan-2-yl)oxymethyl]-6-(3,4,5-trimethoxyphenoxy)oxane-3,4,5-triol | C_20_H_30_O_13_ | [M+Na]^+^ | 5.1564 | 501.1579 | 501.1569 | -2 | 185.0818, 153.0553, 154.0625, 227.0937, 209.0813 | Positive | 2 | LZ、P |
| 282 | (2R,3S,4S,5R,6S)-2-(hydroxymethyl)-6-(3,4,5-trimethoxyphenoxy)oxane-3,4,5-triol | C_15_H_22_O_9_ | [M+H]^+^ | 5.1375 | 347.1337 | 347.1317 | -5.76 | 153.0554, 185.0820, 154.0618, 125.0596, 139.0395 | Positive | 2 | P |
| Alkaloids and their derivatives | | | | | | | | | | | |
| 283 | Uric Acid | C_5_H_4_N_4_O_3_ | [M-H]^-^ | 5.9887 | 167.0211 | 167.0205 | -3.59 | 124.0151, 96.0200, 69.0092, 97.0040, 142.0256 | Negative | 2 | LZ、P |
| 284 | Riboflavin | C_17_H_20_N_4_O_6_ | [M+H]^+^ | 1.6199 | 377.1456 | 377.1449 | -1.86 | 172.0869, 198.0659, 243.0874, 200.0814, 170.0720 | Positive | 2 | MZ |
| 285 | Phenylethanolamine | C_8_H_11_NO | [M+H]^+^ | 5.621 | 138.0913 | 138.0910 | -2.17 | 77.0400, 51.0241, 91.0559, 65.0400, 103.0560 | Positive | 2 | LZ |
| 286 | Norharman | C_11_H_8_N_2_ | [M+H]^+^ | 14.2226 | 169.0760 | 169.0757 | -1.77 | 115.0562, 115.0509, 89.0359, 140.0448, 141.0558 | Positive | 2 | LZ |
| 287 | N-Cyclohexanecarbonylpentadecylamine | C_22_H_43_NO | [M-H]^-^ | 6.7261 | 245.0932 | 245.0939 | 2.86 | 121.1013, 135.1167, 81.0704, 93.0703, 95.0859 | Negative | 2 | LZ |
| 288 | N-Acetyltryptophan | C_13_H_14_N_2_O_3_ | [M+H]^+^ | 5.9708 | 247.1077 | 247.1085 | 3.24 | N/A | Positive | 2 | LZ |
| 289 | N6-(Delta2-isopentenyl)-adenine | C_10_H_13_N_5_ | [M+H]^+^ | 1.5843 | 204.1244 | 204.1247 | 1.47 | 136.0610, 119.0356, 148.0599, 69.0714, 92.0254 | Positive | 2 | LZ |
| 290 | N,N-dimethyl-proline-proline betaine | C_12_H_20_N_2_O_3_ | [M+H]^+^ | 4.6856 | 241.1547 | 241.1549 | 0.83 | 58.0670, 196.0960, 84.0764, 128.1048, 70.0665 | Positive | 2 | LZ、P |
| 291 | Higenamine | C_16_H_17_NO_3_ | [M+H]^+^ | 5.931 | 272.1281 | 272.1284 | 1.1 | 107.0490, 161.0590, 143.0490, 115.0540, 123.0440 | Positive | 2 | LZ、MZ、P |
| 292 | Harmane | C_12_H_10_N_2_ | [M+H]^+^ | 6.6474 | 183.0917 | 183.0918 | 0.55 | 115.0547, 168.0693, 116.0578, 182.0839, 142.0662 | Positive | 2 | LZ、P |
| 293 | Dibenzylamine | C_14_H_15_N | [M+H]^+^ | 6.5716 | 198.1277 | 198.1278 | 0.5 | 91.0542, 65.0385, 165.0699, 106.0651, 79.0542 | Positive | 2 | LZ、MZ、P |
| 294 | allantoin | C_4_H_6_N_4_O_3_ | [M+Na]^+^ | 3.4288 | 181.0332 | 181.0340 | 4.42 | 152.0620, 153.0700, 92.0250, 120.0200, 108.0200 | Positive | 2 | LZ、MZ、P |
| 295 | 4-Quinolinecarboxylic acid | C_10_H_7_NO_2_ | [M+H]^+^ | 18.292 | 174.0550 | 174.0548 | -1.15 | 130.0645, 146.0592, 77.0406, 128.0487, 103.0549 | Positive | 2 | MZ |
| 296 | 3.10S-Hydroxypheophorbide a | C_35_H_36_N_4_O_5_ | [M+H]^+^ | 1.5811 | 609.2707 | 609.2716 | 1.48 | 531.2397, 591.2614, 559.2347, 515.2449, 485.2349 | Positive | 2 | MZ |
| 297 | 1-Methyl-6,7-dihydroxy-1,2,3,4-tetrahydroisoquinoline | C_10_H_13_NO_2_ | [M+H]^+^ | 5.3645 | 180.1019 | 180.1019 | 0 | 115.0546, 91.0559, 145.0645, 117.0697, 163.0749 | Positive | 2 | LZ、P |
| 298 | (E)-N-(4-acetamidobutyl)-3-(4-hydroxy-3-methoxyphenyl)prop-2-enamide | C_16_H_22_N_2_O_4_ | [M+H]^+^ | 5.0082 | 307.1652 | 307.1766 | 2.6 | 145.0283, 177.0545, 117.0332, 149.0594, 89.0388 | Positive | 2 | LZ、P |
| 299 | Eleutherazine B | C_22_H_36_N_4_O_6_ | [M+Na]^+^ | 1.2269 | 475.2527 | 475.2520 | -1.47 | 445.2413, 415.2308, 476.2554, 446.2449 | Positive | 2 | LZ、P |

**Supplementary Table S2.** Parameters of OPLS-DA models.

| OPLS-DA model | R2X(cum) | R2Y(cum) | Q2(cum) | P |
| --- | --- | --- | --- | --- |
| LZvsMZ | 0.506 | 0.986 | 0.855 | <0.05 |
| MZvsP | 0.461 | 0.913 | 0.747 | <0.05 |
| LZvsP | 0.433 | 0.892 | 0.767 | <0.05 |

**Supplementary Table S3.** Metabolites with significantly increased accumulation in the LZ group among differential compounds.

| Peak.NO | Metabolite | Ion | VIP | P | Log2FC |
| --- | --- | --- | --- | --- | --- |
| 1 | feruloyltyramine | - | 1.410173091 | 0.030286 | 1.3472 |
| 2 | 5-hydroxy-2-(4-hydroxyphenyl)-7-methoxy-2,3-dihydrochromen-4-one | + | 1.611899318 | 0.0032036 | 3.3252 |
| 3 | 2,3-bis[(4-hydroxy-3-methoxyphenyl)methyl]butane-1,4-diol | + | 1.514166294 | 0.0045935 | 2.2723 |
| 4 | Isoliquiritigenin | + | 2.367660064 | 3.8708e-06 | 3.3777 |
| 5 | 6-desmethyl-sideroxylin | + | 1.125276831 | 0.045553 | 1.7712 |
| 6 | pinocembrin | + | 1.46676154 | 0.017644 | 1.8832 |
| 7 | Secoisolariciresinol | + | 1.551993947 | 0.0082713 | 2.5785 |
| 8 | 7-Hydroxyflavanone | + | 1.495287061 | 0.021995 | 3.4667 |
| 9 | linoleic acid | - | 1.306237976 | 0.023146 | 2.3516 |
| 10 | sucrose | + | 1.340580551 | 0.025171 | 1.492 |
| 11 | Palatinose | + | 1.561607303 | 0.010479 | 1.3565 |
| 12 | 9-Oxo-10E,12Z-octadecadienoic acid | + | 1.35722056 | 0.014902 | 1.1425 |
| 13 | alpha-Bisabolol | - | 1.582154673 | 0.0025926 | 2.0439 |
| 14 | 1-Methyl-6,7-dihydroxy-1,2,3,4-tetrahydroisoquinoline | - | 1.272354753 | 0.019719 | 3.9158 |
| 15 | 3-[(E)-2-(3-hydroxyphenyl)ethenyl]-5-methoxyphenol | + | 1.303198469 | 0.044295 | 1.1897 |
| 16 | Sphinganine | + | 1.193287365 | 0.033655 | 3.1573 |
| 17 | 5-[6-(3-hydroxy-4-methoxyphenyl)-1,3,3a,4,6,6a-hexahydrofuro[3,4-c]furan-3-yl]-2-methoxyphenol | + | 1.357043405 | 0.018021 | 1.6989 |
| 18 | Apigenin | - | 1.243648566 | 0.033104 | 2.0683 |
| 19 | Melibiose | + | 1.315831974 | 0.02809 | 1.9054 |
| 20 | 4-[(3R,3aR,6S,6aR)-6-(4-hydroxy-3,5-dimethoxyphenyl)-1,3,3a,4,6,6a-hexahydrofuro[3,4-c]furan-3-yl]-2,6-dimethoxyphenol | + | 1.462188486 | 0.010379 | 1.8161 |
| 21 | Paprazine | + | 1.299106707 | 0.022439 | 1.6543 |
| 22 | PC(0:0/18:1) | + | 1.547524816 | 0.0056763 | 1.5252 |
| 23 | Sinapyl alcohol | - | 1.628144516 | 0.0023268 | 3.232 |
| 24 | 12(13)-Epoxy-9Z-octadecenoic acid | - | 1.224170205 | 0.025178 | 3.0535 |
| 25 | Azelaic acid | - | 1.67578682 | 0.0028421 | 1.5405 |
| 26 | Sorbitol | - | 1.342163563 | 0.030976 | 1.4885 |
| 27 | Pinoresinol | - | 1.94736239 | 0.0023303 | 1.7366 |
| 29 | 7-O-Methylchrysin | + | 1.6880081 | 0.0013462 | 3.3018 |
| 30 | (E)-N-(4-acetamidobutyl)-3-(4-hydroxy-3-methoxyphenyl)prop-2-enamide | + | 1.477670076 | 0.0068697 | 3.8661 |
| 31 | 2-Methoxycinnamaldehyde | + | 1.802605038 | 0.00046829 | 3.0518 |
| 32 | Vanillic acid | - | 1.400447641 | 0.016567 | 1.4361 |
| 33 | Globulol | - | 1.463338596 | 0.0079646 | 2.0828 |
| 34 | N-Oleoylethanolamine | + | 1.537245893 | 0.0043813 | 3.4423 |
| 35 | Syringaldehyde | + | 1.640870312 | 0.0020857 | 2.8531 |
| 36 | Phytoceramide C2 | + | 1.217779143 | 0.02789 | 2.617 |
| 38 | oleic acid | + | 1.871994856 | 0.00024965 | 1.9473 |
| 39 | 3,4,5-Trimethoxyphenol | + | 1.666638405 | 0.0011511 | 2.9548 |
| 40 | isolariciresinol | - | 1.824827529 | 0.0011053 | 4.544 |
| 41 | Glycerol 1-stearate | + | 1.966767025 | 0.00014621 | 1.5911 |
| 42 | PC(19:1/0:0) | + | 1.32912867 | 0.020597 | 1.848 |
| 43 | 1-Naphthol | + | 1.26219809 | 0.021851 | 1.1844 |
| 44 | Syringic acid | + | 1.695788036 | 0.0011781 | 1.8405 |
| 45 | Citric acid | - | 1.214730142 | 0.042159 | 3.7327 |
| 46 | 3,3'-Dihydroxyflavone | - | 1.413078082 | 0.021504 | 1.6648 |
| 47 | 4-O-Methylphloracetophenone | - | 1.29306674 | 0.046108 | 2.5106 |
| 48 | (2R,3S,4S,5R,6S)-2-[(3,4,5-trihydroxyoxan-2-yl)oxymethyl]-6-(3,4,5-trimethoxyphenoxy)oxane-3,4,5-triol | + | 1.166074266 | 0.049391 | 2.6576 |
| 49 | Monoolein | + | 1.416738023 | 0.0098289 | 1.9481 |
| 50 | 6,7-bis(hydroxymethyl)-1-methoxy-8-(3,4,5-trimethoxyphenyl)-5,6,7,8-tetrahydronaphthalene-2,3-diol | + | 1.276367125 | 0.022788 | 2.4498 |

**Supplementary Table S4.** Metabolites with significantly increased accumulation in the MZ group among differential compounds.

| Peak.NO. | Metabolite | Ion | VIP | P | Log2FC |
| --- | --- | --- | --- | --- | --- |
| 1 | 1-({[2-(2-furylmethyl)-5-methylpyrrolidinyl]amino}methylene)-7-[8-({[2-(2-fury lmethyl)pyrrolidinyl]amino}methylene)-1,6-dihydroxy-3-methyl-5-(methylethyl)-7 -oxo(2-naphthyl)]-3,8-dihydroxy-6-methyl-4-(methylethyl)naphthalen-2-one | + | 1.29095819 | 0.0039256 | 5.0367 |
| 2 | 8-desmethyl-sideroxylin | + | 1.056834709 | 0.048888 | 6.8473 |
| 3 | ruscogenin | + | 1.530253914 | 0.00056263 | 5.9455 |
| 4 | 4-methoxy-3-[(4-methoxyphenoxy)methyl]benzaldehyde | + | 1.395825745 | 0.0094855 | 1.0618 |
| 5 | Alpinetin | + | 1.067827932 | 0.023316 | 1.3883 |
| 6 | 12-Oxodihydrophytodienoic acid | + | 1.614065701 | 0.00029019 | 6.8927 |
| 7 | 5,4'-Dihydroxy-7-methoxyflavanone | + | 1.280157246 | 0.025739 | 4.4288 |
| 8 | gamma-linolenic acid | + | 1.336299752 | 0.0047276 | 4.6871 |
| 9 | Apigeninidin | + | 1.294212323 | 0.010527 | 4.0354 |
| 10 | Chrysin | + | 1.492967665 | 0.011876 | 1.9798 |
| 11 | Sinapic acid | + | 1.276760279 | 0.0060281 | 3.1053 |
| 12 | Tangeretin | + | 1.035885969 | 0.034237 | 3.1153 |
| 13 | 4'-Hydroxy-7-methoxyflavone | + | 1.409000321 | 0.026405 | 3.3719 |
| 14 | Pinoresinol | - | 1.204833781 | 0.035622 | 1.2612 |
| 15 | 1-(9Z-Octadecenoyl)-sn-glycero-3-phosphocholine | + | 1.559768997 | 0.00068976 | 3.2311 |
| 16 | Umbelliferone | - | 2.081412632 | 0.00012694 | 4.2453 |
| 17 | 2,6-Di-tert-butyl-4-hydroxymethylphenol | + | 1.648474419 | 0.002219 | 1.0283 |
| 18 | 7,8-Dihydroxycoumarin | - | 1.372263171 | 0.023388 | 1.1152 |
| 19 | cholesta-5,7-dien-3beta-ol | + | 1.529439168 | 0.0019359 | 2.8365 |
| 20 | Cholesterol | + | 1.569361418 | 0.0047139 | 1.0527 |
| 21 | cis,cis-9,12-Octadecadien-1-ol | + | 1.022784722 | 0.039632 | 1.1834 |
| 22 | 3,3',4'-Trimethoxyflavone | + | 1.388897752 | 0.014629 | 2.4652 |
| 23 | 4,7-Methanoazulen-6(1H)-one, 2,4,5,7,8,8a-hexahydro-7-hydroxy-9-(hydroxymethyl)-1,4,9-trimethyl-, (1S,4R,7R,8aS)- | + | 1.074884888 | 0.049883 | 1.8166 |
| 24 | 2,6-Dimethoxybenzoic acid | + | 1.109051813 | 0.049856 | 1.7465 |
| 25 | Arenamide A | + | 1.038080341 | 0.048874 | 1.2073 |
| 26 | [3,4,5-trihydroxy-6-(hydroxymethyl)oxan-2-yl] 2,4-dihydroxy-6-[(E)-2-phenylethenyl]benzoate | + | 1.17857261 | 0.042272 | 1.6132 |
| 27 | 5,6,7,8-tetramethoxy-2-(4-methoxyphenyl)chromen-4-one | + | 1.245093794 | 0.027915 | 1.4791 |
| 28 | 8-{(1S,5R)-4-Oxo-5-[(2Z)-2-penten-1-yl]-2-cyclopenten-1-yl}octanoic acid | + | 1.316669859 | 0.021207 | 1.4903 |
| 29 | Lactulose | + | 1.114683012 | 0.046956 | 1.1757 |
| 30 | Homovanillic acid | + | 1.29047086 | 0.025349 | 2.9808 |
| 31 | Kaempferol-7-O-rhamnoside | + | 1.038080341 | 0.048874 | 1.2073 |
| 32 | 5-phenyl-1,3-oxazolidin-2-one | + | 1.508731511 | 0.0046728 | 1.0305 |
| 33 | Eupatorin | + | 1.038080341 | 0.048874 | 1.2073 |
| 34 | (+/-)-Taxifolin | + | 1.245093794 | 0.027915 | 1.4791 |
| 35 | PE(16:0/18:1) | + | 1.616924153 | 0.010677 | 1.231 |
| 36 | 1-Heptadecanoyl-sn-glycero-3-phosphocholine | + | 1.038080341 | 0.048874 | 1.2073 |
| 37 | Stachyose | + | 1.114683012 | 0.046956 | 1.1757 |
| 38 | Riboflavin | + | 1.038080341 | 0.048874 | 1.2073 |
| 39 | 10S-Hydroxypheophorbide | + | 2.3091 | 1.2073 | 0.048874 |
| 40 | Xefoampeptide E | + | 1.508731511 | 0.0046728 | 1.0305 |
| 41 | 3,4,5-Trimethoxybenzaldehyde | + | 1.569361418 | 0.0047139 | 1.0527 |
| 42 | Adenosine | + | 1.508731511 | 0.0046728 | 1.0305 |

Supplementary Table S5. Metabolites with significantly increased accumulation in the P group among differential compounds.

| Peak.NO | Metabolite | Ion | VIP | P | Log2FC |
| --- | --- | --- | --- | --- | --- |
| 1 | 4',7-Dihydroxyflavone | + | 1.660122486 | 0.0017481 | 1.9222 |
| 2 | Moupinamide | + | 1.32815706 | 0.011996 | 2.0372 |
| 3 | trans-pterostilbene | + | 2.538189577 | 0.00092356 | 2.7211 |
| 4 | 3-{2,4-dihydroxy-5-[3-(4-hydroxy-2-methoxyphenyl)-1-(4-hydroxyphenyl)propyl]phenyl}-1-(4-hydroxyphenyl)propan-1-one | + | 1.687782488 | 0.040355 | 1.0876 |
| 5 | Biochanin | + | 1.695508575 | 0.043399 | 1.5727 |
| 6 | Isoliquiritigenin | + | 1.869921365 | 0.0091943 | 1.0816 |
| 7 | 2,4-Dimethoxy-2'-hydroxychalcone | + | 2.160805293 | 0.0016159 | 1.6339 |
| 8 | 3-[(E)-2-(3-hydroxyphenyl)ethenyl]-5-methoxyphenol | - | 2.150987039 | 0.00027632 | 1.5397 |
| 9 | Eleutheroside E | + | 1.886015819 | 0.035581 | 2.0042 |
| 10 | 7-hydroxy-2-(4-hydroxyphenyl)-5-methoxy-2,3-dihydro-4h-chromen-4-one | + | 1.448955132 | 0.0054853 | 1.4887 |
| 11 | sucrose | + | 1.300487983 | 0.037345 | 1.9887 |
| 12 | (S)-3-(4-hydroxyphenyl)chroman-7-ol | + | 1.419571623 | 0.00014362 | 1.7904 |
| 13 | 5-hydroxy-2-(4-hydroxyphenyl)-7-methoxy-2,3-dihydrochromen-4-one | + | 1.317862825 | 0.013817 | 2.4836 |
| 14 | Episyringaresinol 4'-O-beta-D-glncopyranoside | + | 1.538957111 | 0.0076988 | 1.7278 |
| 15 | linolenic acid | + | 1.066279888 | 0.045393 | 1.3398 |
| 16 | (E)-3-(3,4-dihydroxy-2-methoxyphenyl)-1-(4-hydroxyphenyl)prop-2-en-1-one | + | 1.585942691 | 0.029149 | 1.8831 |
| 17 | Paprazine | + | 1.953450503 | 0.037119 | 1.4413 |
| 18 | 2,3-bis[(4-hydroxy-3-methoxyphenyl)methyl]butane-1,4-diol | + | 1.251365958 | 0.030044 | 1.2076 |
| 19 | 4-[(3R,3aR,6S,6aR)-6-(4-hydroxy-3,5-dimethoxyphenyl)-1,3,3a,4,6,6a-hexahydrofuro[3,4-c]furan-3-yl]-2,6-dimethoxyphenol | + | 1.884515006 | 0.00025291 | 2.2252 |
| 20 | 7-Hydroxyflavanone | + | 1.408141506 | 0.018119 | 3.541 |
| 21 | Dibenzylamine | + | 1.627870857 | 0.015045 | 2.1131 |
| 22 | Coniferaldehyde | + | 1.289333554 | 0.022284 | 1.0707 |
| 23 | Genkwanin | + | 1.430061255 | 0.01006 | 1.8889 |
| 24 | linoleic acid | + | 1.190540899 | 0.024021 | 1.2247 |
| 25 | Syringaldehyde | + | 1.835366373 | 0.00025519 | 2.3015 |
| 26 | 5,4'-Dihydroxy-7-methoxyflavanone | + | 1.835366373 | 0.00025519 | 2.3015 |
| 27 | Pinocembrine | + | 1.226865412 | 0.010359 | 1.9585 |
| 28 | Sphinganine | + | 1.52518719 | 0.0077726 | 1.6532 |
| 29 | 7-O-Methylchrysin | + | 1.242118786 | 0.020723 | 2.801 |
| 30 | Sinapoyl aldehyde | - | 1.256989539 | 0.044912 | 1.1491 |
| 31 | 3,4,5-Trimethoxyphenol | + | 1.761411233 | 0.00054203 | 2.7981 |
| 32 | 2-Methoxycinnamaldehyde | + | 1.022551488 | 0.025546 | 2.6236 |
| 33 | 4-Allyl-2-(beta-D-glucopyranosyloxy)phenyl beta-D-glucopyranoside | + | 1.201424639 | 0.046095 | 1.3519 |
| 34 | Glycerol 1-stearate | + | 1.57413845 | 0.00059618 | 1.5415 |
| 35 | PC(19:1/0:0) | + | 1.439465561 | 0.0029691 | 1.8529 |
| 36 | (2S,3R,4S,5S,6R)-2-[4-[(3S,3aR,6S,6aR)-3-(4-hydroxy-3,5-dimethoxyphenyl)-1,3,3a,4,6,6a-hexahydrofuro[3,4-c]furan-6-yl]-2,6-dimethoxyphenoxy]-6-(hydroxymethyl)oxane-3,4,5-triol | + | 1.67502622 | 0.0037582 | 2.6455 |
| 37 | 1-Naphthol | + | 1.578403722 | 0.0027306 | 1.6388 |
| 38 | 1-Methyl-6,7-dihydroxy-1,2,3,4-tetrahydroisoquinoline | + | 1.580845637 | 0.00483 | 1.4289 |
| 39 | N-Oleoylethanolamine | + | 1.663579941 | 0.0030576 | 1.928 |
| 40 | 2-Phenylethyl 6-O-(6-deoxy-alpha-L-mannopyranosyl)-beta-D-glucopyranoside | + | 1.249590317 | 0.046163 | 1.6472 |
| 41 | 3',5'-Dimethoxy-4'-hydroxyacetophenone | + | 1.603867526 | 0.00050679 | 2.3871 |
| 42 | beta-D-Glucopyranoside, 2-hydroxy-4-(2-propen-1-yl)phenyl 6-O-beta-D-glucopyranosyl- | + | 1.386370503 | 0.017541 | 2.2671 |
| 43 | N,N-dimethyl-proline-proline betaine | + | 1.799560061 | 0.0021445 | 2.5815 |
| 44 | Raffinose | + | 1.228083224 | 0.045543 | 1.1566 |
| 45 | Chrysin | + | 2.218358935 | 0.0022053 | 2.6038 |
